# First use of a divalent lanthanide for visible-light-promoted photoredox catalysis†
†Electronic supplementary information (ESI) available. CCDC 1539923. For ESI and crystallographic data in CIF or other electronic format see DOI: 10.1039/c7sc02479g


**DOI:** 10.1039/c7sc02479g

**Published:** 2017-12-21

**Authors:** Tyler C. Jenks, Matthew D. Bailey, Jessica L. Hovey, Shanilke Fernando, Gihan Basnayake, Michael E. Cross, Wen Li, Matthew J. Allen

**Affiliations:** a Department of Chemistry , Wayne State University , 5101 Cass Avenue , Detroit , MI 48202 , USA . Email: mallen@chem.wayne.edu

## Abstract

We report the first catalytic use of a divalent lanthanide in visible-light-promoted bond-forming reactions. Our new precatalyst uses europium in the +2 oxidation state and is active in the presence of blue light from light-emitting diodes. The use of low-energy visible light reduces the occurrence of potential side reactions that might be induced by higher-energy UV light. The system described here uses zinc metal as a sacrificial reductant and is tolerant to wet, protic solvents. The catalyst can be made *in situ* from relatively inexpensive and air-stable EuCl_3_·6H_2_O, and the ligand can be synthesized in large quantities in two steps. With 0.5% loading of precatalyst, an average of 120 turnovers was observed in six hours for reductive coupling of benzyl chloride. We expect that the results will initiate the study of visible-light-promoted photoredox catalysis using divalent europium in a variety of reactions.

## Abstract 2

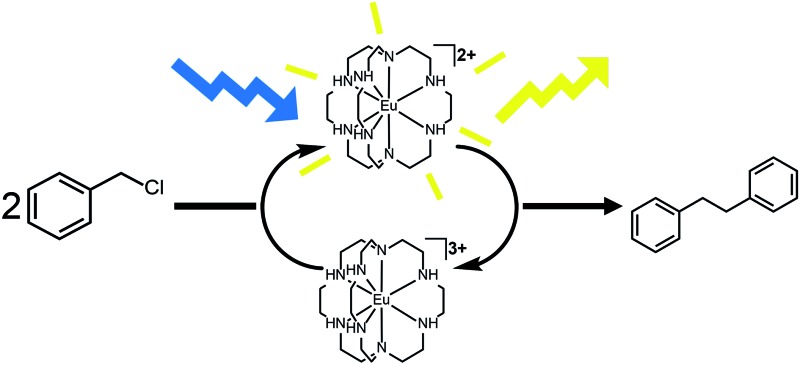
Divalent europium is used catalytically in visible-light-promoted photoredox bond-forming reactions.

## Introduction

Metal-assisted photoredox catalysis uses light to promote the reactivity of metal-containing complexes in reactions such as halogen-atom abstractions, functional-group reductions, and carbon–carbon bond formations.^1^–^3^ Most reported metal-assisted photoredox systems rely on transition metals,^2^ with a small number of photoredox systems involving lanthanides that are either catalytic *via* the +3/+4 redox couple^3^–^5^ or noncatalytic starting from the +2 oxidation state.^6^–^8^ Among these metals, Eu^II^ is unique in that it is the mildest reducing agent of the divalent lanthanides. It can be handled in protic solvents including water; it can be produced from Eu^III^, which is inexpensive relative to second and third row transition metals commonly used in photoredox catalysis; and it undergoes metal–orbital-based electronic transitions that are not susceptible to photobleaching like organic dyes.^9^ Recently, we reported a luminescent, aqueous, Eu^II^-containing complex that had a high quantum yield (26%) for a 5d–4f transition that occurred in the visible region of the electromagnetic spectrum using a ligand that can be prepared on large scale in two steps.^10^,^11^ We hypothesized that because this complex is luminescent and contains a redox-active metal, it could be employed in photoredox reactions with a sacrificial reducing agent to make the reaction catalytic in europium. Here, we report the first catalytic example of carbon–carbon bond formation using a europium-containing complex and visible light. Further, we evaluate the mechanism of the catalytic system.

## Results and discussion

**Fig. 1:**
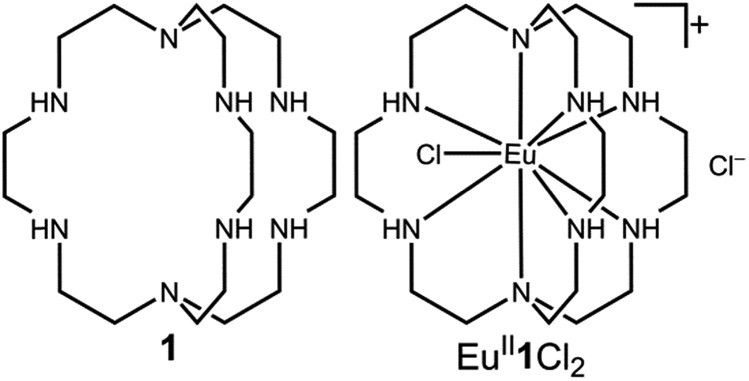
Structures of ligand **1** (left) and Eu^II^**1** (right).

Our photoredox system relies on azacryptand 1,4,7,10,13,16,21,24-octaazabicyclo[8.8.8]hexacosane, **1**, to encapsulate Eu^II^, inducing a bathochromic shift in the UV-visible absorption of Eu^II^ from the UV to the visible region of the electromagnetic spectrum (Fig. 1). This bathochromic shift arises from d-orbital splitting, caused by the nitrogen atoms of the cryptand, that results in a lower-energy 5d–4f transition relative to transitions induced by weaker field ligands.10*b* Upon absorption of blue light by Eu^II^**1**, an electron is excited into an emissive state that has a luminescence lifetime of 0.98 ± 0.03 μs and a quantum yield of 37% in methanol. The quantum yield of Eu^II^**1** in methanol is 11% higher than the previously reported value for the same complex in a pH 12 aqueous solution,10*a* and the difference in the quantum yield is likely caused by the change of solvent. The luminescence lifetime of Eu^II^**1** is in the range of typical photoredox systems.^12^ Interestingly, both Eu^II^ and Ce^III^ are known to be emissive through 5d–4f transitions with typical lifetimes on the order of 1 ns to 1 μs.^3^,^7^,^13^,^14^ This range of lifetimes for similar electronic transitions suggests that these lifetimes are largely dependent on ligand field and not necessarily intrinsic to the metal ions. The values for lifetime and quantum yield are toward the long and high end, respectively, of reports for solvated Eu^II^.^15^,^16^ Due to the photophysical properties of Eu^II^**1**, including the efficient conversion of visible light to a long-lived excited state, we hypothesized that Eu^II^**1** would be a good promoter of photoredox reactions.

**Fig. 2:**
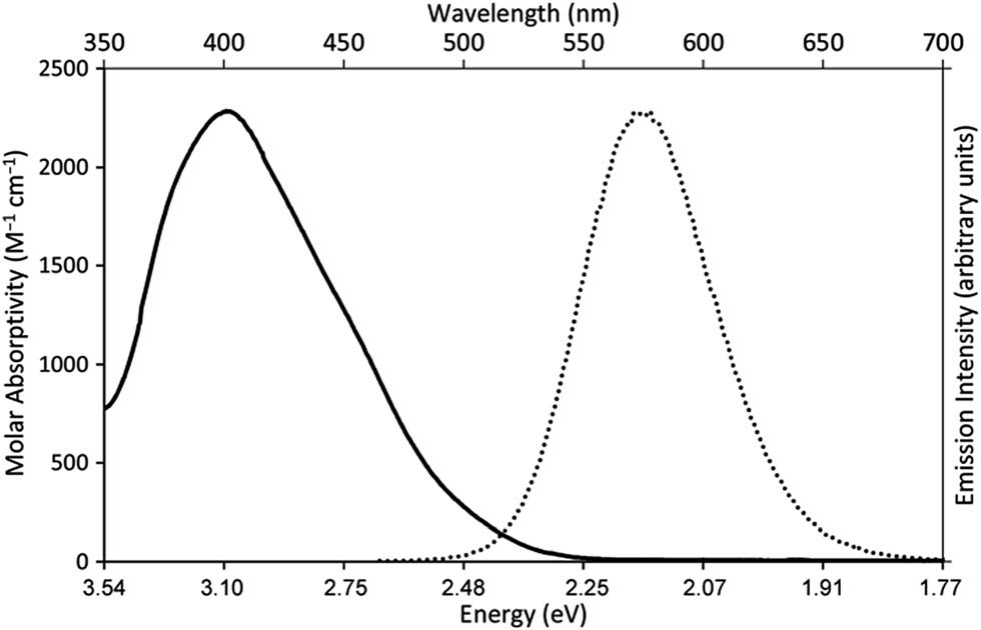
UV-visible absorption spectrum of Eu^II^**1**Cl_2_ (—, left *y*-axis) and emission spectrum (*λ*_ex_ = 460 nm, *ε*: 1044 M^–1^ cm^–1^) of Eu^II^**1**Cl_2_ (••, right *y*-axis). Spectra were acquired in methanol.

When a redox-active metal complex is excited to an emissive state, the *E*_1/2_ of the complex changes.^1^–^6^ To estimate the *E*_1/2_ of Eu^II^**1** in the emissive state, the excited-state potential 
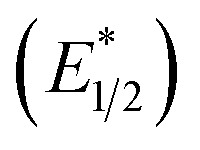
 was calculated by means of the Rehm–Weller formalism (eqn (1)) using the ground-state potential (*E*_1/2_) and the energy of the emission band (*E*_0,0_), which is the energy of an electron in the excited state relative to the ground state as determined by the maximum emission wavelength (Fig. 2).^17^ There is an additional work-function term that has been omitted from eqn (1) because it was assumed to be negligibly small.^4^ To determine the ground-state potential of Eu^II^**1**, cyclic voltammetry was performed with Eu^II^**1** in *N*,*N*-dimethylformamide. A reversible Eu^II/III^**1** couple was observed with an *E*_1/2_ of –0.90 V *vs.* Ag/AgCl, which represents a negative shift in the *E*_1/2_ potential relative to the solvated Eu^II/III^ couple, and the negative shift is consistent with other reported Eu^II^ complexes that contain nitrogen donors.^18^,^19^ *E*_0,0_ was estimated to be 2.14 V by dividing the product of Planck's constant and the speed of light by the maximum emission wavelength (580 nm) in meters (*hc*/*λ*). Using these values for the ground-state potential and the emission-band energy, the 
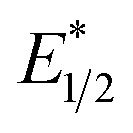
 of Eu^II^**1** was calculated to be –3.0 V *vs.* Ag/AgCl. This calculated excited-state potential is among the most negative excited-state potentials reported to date for metal-based catalytic photoredox agents and is more negative than the potential of the potent reducing agent SmI_2_ in the presence of hexamethylphosphoramide.^20^,^21^ With a sense of the redox properties of Eu^II^**1** in hand, we were interested in probing the reactivity of Eu^II^**1**. On the basis of a recent report from the Schelter group describing photocatalytic reductive couplings using a Ce^III/IV^ system,^3^ we expected that Eu^II^**1** would display similar reactivity.1
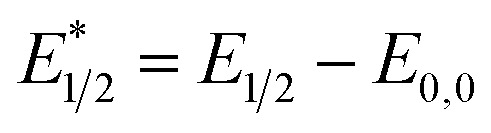



**Fig. 3:**
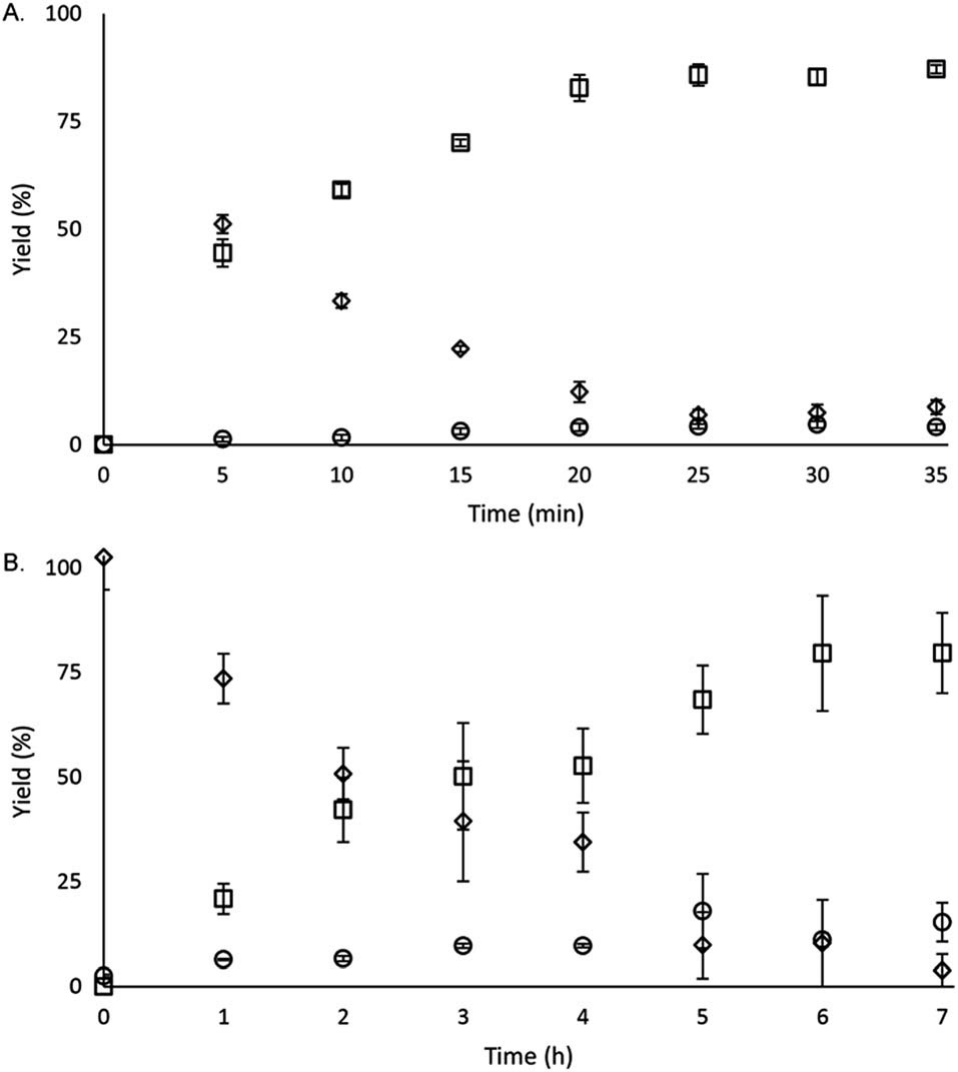
Formation of products and disappearance of starting material as a function of time for (A) stoichiometric and (B) catalytic (10 mol%) benzyl chloride coupling reactions (squares = 1,2-diphenylethane, diamonds = benzyl chloride, and circles = toluene). Each point is the mean of three independently prepared reactions, and the error bars represent the standard error of the means.

To study the reactivity of Eu^II^**1**, we attempted to reductively couple alkyl halides to form carbon–carbon bonds. A solution containing EuCl_2_ (1 equiv.), **1** (1 equiv.), and benzyl chloride (1 equiv., 0.027 mmol) in methanol was illuminated with blue light (∼7.6 W, *λ*_em_ = 460 nm, Fig. S2†) using a strip of light-emitting diodes. We observed the formation of 1,2-diphenylethane (85 ± 2%) and toluene (4.7 ± 0.4%) within 30 minutes (Fig. 3A).^22^

To determine whether the reaction was promoted by the excited-state of Eu^II^**1**, we performed three control reactions (Table 1). When the coupling of benzyl chloride was attempted in the absence of light, no product was observed. This observation indicated that for the reaction to proceed, light must be present, suggesting that the excited state of Eu^II^**1** was promoting the reaction and not the ground state of Eu^II^**1**. When ligand **1** was omitted, no product was observed. This observation indicated that uncomplexed europium ions are incapable of performing the reductive coupling. When EuCl_2_ was omitted, no product was observed, indicating that europium is an active participant in the reduction of benzyl chloride. The control reactions demonstrate that light, ligand **1**, and europium are all necessary to reduce benzyl chloride. To test for reactivity with methanol, fluorescence spectroscopy was performed before and after 12 h of light exposure on samples of Eu^II^**1** (Fig. S20†). Based on these studies, the excited state of Eu^II^**1** reacts with methanol, but no reaction with methanol was observed over the same time period in the dark. Despite the reactivity of the excited state of Eu^II^**1** with methanol, the observation of 1,2-diphenylethane in excellent yields in 30 min indicates that the reaction with methanol is relatively slow. To further understand how Eu^II^**1** promotes light-induced bond formation, we attempted to determine the mechanism of electron transfer.

**Table 1:** Stoichiometric control reactions

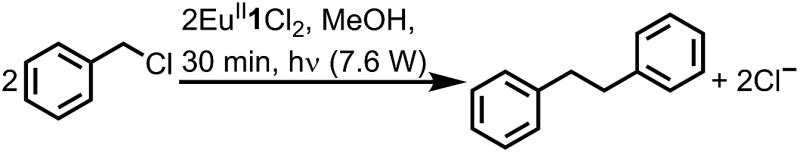	
Conditions	Yield^*a*^
Unmodified	85 ± 2%
Dark	No reaction
No **1**	No reaction
No Eu	No reaction

^*a*^Determined by gas chromatography-mass spectrometry.

The emissive state of Eu^II^**1** is responsible for the observed reactivity, and it is unlikely that energy transfer occurs between the emissive state of Eu^II^**1** and benzyl chloride as shown by the lack of spectral overlap between the absorption of benzyl chloride and the emission of Eu^II^**1**; therefore, the reductive coupling of benzyl chloride must occur through a photoinduced electron transfer, which would be expected to quench luminescence. We sought to investigate the mechanism of photoinduced electron transfer using substrates to quench luminescence with Stern–Volmer analyses.^23^ We measured the rate of quenching (*k*_q_) of the excited-state intensity (*I*) as function of concentration of substrates (Table 2). Additionally, we measured *k*_q_ at three different temperatures for benzyl chloride and attempted to obtain lifetime quenching data. Entries 1 and 2 showed no detectable quenching of luminescence with Eu^II^**1**, unlike entries 3 and 4 (Table 2). For entries 3 and 4, plots of *I*_0_/*I versus* concentration of quencher resulted in the observation of linear relationships (Fig. S15†). The linear relationships are indicative of well-behaved bimolecular quenching interactions that can be either collisional or static in nature.^23^ Furthermore, *k*_q_ increased with increasing temperature, suggesting that the quenching is likely due to a diffusion-limited, collisional mechanism and is not static in nature (Fig. S16†). The collisional mechanism eliminates the possibility of the participation of a preorganized benzyl chloride adduct of Eu^II^**1** in the reaction. These results are consistent with the reaction of benzyl bromide with divalent europium in the presence of crown ethers.^7^ In both cases, the values of *k*_q_ differ from the idealized collisional bimolecular quenching constant (10^10^ M^–1^ s^–1^).^23^ These differences are likely due to coordinative saturation of Eu^II^, causing a lower frequency of productive collisions between Eu^II^ and substrates compared to idealized lumophores.

**Table 2:** Stern–Volmer data

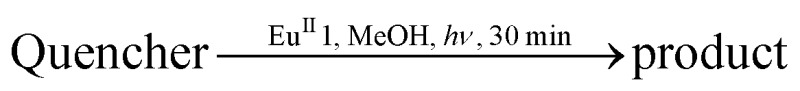					
Entry	Quencher	*E*_pc_ of quencher (V *vs.* Ag/AgCl)	*k*_q_ (×10^7^ M^–1^ s^–1^)	Product	Yield^*a*^ (%)
1	(CH_3_)_3_CCl	–3.05	0^*b*^	[(CH_3_)_3_C]_2_	1.9 ± 0.1
2	C_6_H_5_Cl	–2.93	0^*b*^	C_6_H_6_	5.4 ± 0.4
3	CH_2_CHCH_2_Cl	–2.35	8.5	(CH_2_CHCH_2_)_2_	46 ± 2
4	C_6_H_5_CH_2_Cl	–2.34	73	(C_6_H_5_CH_2_)_2_	85 ± 2

^*a*^Determined by gas chromatography-mass spectrometry.

^*b*^No quenching of the excited state was observed.

To explain the apparent selectivity observed in the Stern–Volmer analyses, cyclic voltammetry was performed for the complex and substrates (Table 2). The peak cathodic potentials of the substrates that showed no quenching of luminescence (*E*_pc_ of entries 1 and 2 in Table 2) are close to or more negative than the calculated 
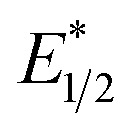
 of Eu^II^**1**. Because reliable cyclic voltammery of Eu^II^**1** could not be obtained in methanol, the *E*_1/2_ of Eu^II^**1** recorded in *N*,*N*-dimethylformamide might have resulted in a more negative value of *E*_1/2_ than would be present in methanol, propagating to a more negative estimation of 
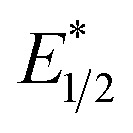
. However, the *E*_pc_ of the substrates that quenched the luminescence of the excited state of Eu^II^**1** (entries 3 and 4 in Table 2) are between the calculated 
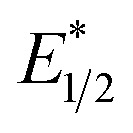
 and ground-state *E*_1/2_ of Eu^II^**1**, consistent with the difference in reactivity of Eu^II^**1** with benzyl chloride in the light and dark. Furthermore, allyl chloride, which has an *E*_pc_ more positive than the 
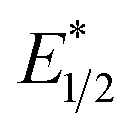
 of Eu^II^**1**, also shows expected product formation in the light (Table 2). Based on the cathodic potentials and lack of observed luminescence quenching, we would not expect chlorobenzene and 2-chloro-2-methylpropane to react with the excited state of Eu^II^**1**; however, products were observed for these two substrates in yields of 1.9 and 5.4%, respectively. These data point toward a thermodynamic window of selectivity (–0.9 to approximately –3 V *vs.* Ag/AgCl) that is unique for Eu^II^1*.

**Fig. 4:**
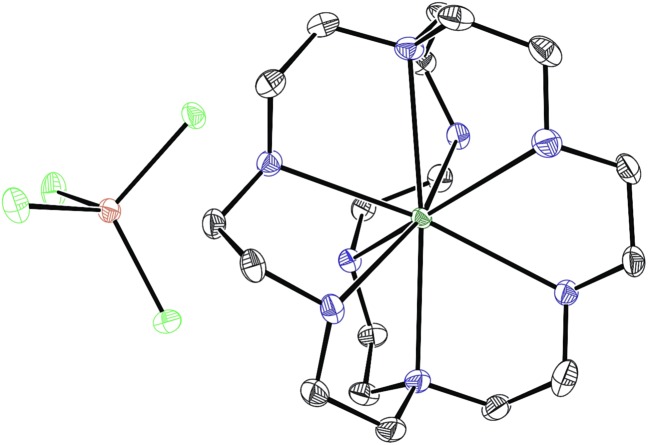
Crystal structure of [Eu^II^**1**][ZnCl_4_] generated from a mixture of EuCl_3_, Zn^0^, and **1** in methanol. Thermal ellipsoids are drawn at 50% probability. Final refinement indicators: *R*_1_ = 2.89%; w*R*_2_ = 6.25%; resolution = 0.4929 Å; *R*_int_ = 4.91%; and *R*_sigma_ = 3.05%. Crystallographic data for this structure has been deposited at the Cambridge Crystallographic Data Centre under deposition number CCDC ; 1539923. An outer-sphere molecule of methanol has been omitted for clarity. Grey = C; blue = N; seagreen = Eu; green = Cl; and brown = Zn.

With an understanding of the electron transfer mechanism of Eu^II^**1**, we were interested in moving from reactions that were stoichiometric in Eu to reactions that were catalytic in Eu. To enable catalysis, a sacrificial reducing agent was needed, and it is known that Eu^III^ can be reduced to Eu^II^*in situ* with Zn^0^.^19^,^24^ To ensure that Eu^II^**1** could be assembled *in situ* from Eu^III^, **1**, and Zn^0^, UV-visible and fluorescence spectroscopies were performed on a mixture of EuCl_3_, Zn^0^, and **1**. Absorption at wavelengths >400 nm and a broad emission between 500 and 700 nm, which are both characteristic of Eu^II^**1**, indicated that Eu^II^**1** can be assembled *in situ* (Fig. S18 and S19†). Furthermore, X-ray diffraction of material nucleated from a mixture of EuCl_3_, Zn^0^, and **1** in methanol provides direct evidence that Eu^II^**1**, as well as oxidized zinc species, are formed under the reaction conditions (Fig. 4). The crystal structure in Fig. 4 is from a crystal isolated from the reaction mixture. Although several crystals formed, a yield was not determined. However, because it nucleated from a reaction mixture in which Eu^II^ was not directly added, this structure demonstrates that Zn^0^ is able to complete the catalytic cycle by either reducing EuCl_3_ followed by metalation with **1** or by reducing Eu^III^**1** to Eu^II^**1**. Direct evidence of the reduction of Eu^III^ to Eu^II^ can be found in the Eu–N bond distances between Eu and the ligand [2.7116(10)–2.7484(10) Å for secondary amines and 2.8030(11)–2.8333(10) Å for tertiary amines] that are in the expected range for Eu^II^–N bonds.10a,^25^ In the structure in Fig. 4, unlike with the previously reported structure of Eu^II^**1**, there was no inner-sphere chloride, and the associated anion was ZnCl_4_^2–^ instead of two equivalents of Cl^–^, indicating oxidation of Zn^0^ and demonstrating the formation of Eu^II^**1***via* reduction of Eu^III^ by Zn^0^.

To ensure that Zn^0^ could not promote the reductive coupling of benzyl chloride, a control experiment was performed with Zn^0^, light, and benzyl chloride. Only the formation of toluene was observed after 6 h, indicating that Zn^0^ does not promote the reductive coupling of benzyl chloride. To probe whether Zn^0^ promoted the formation of toluene, another control experiment was performed that only included benzyl chloride, methanol, and light. This experiment showed no formation of toluene, indicating that Zn^0^ induces the reduction of benzyl chloride to toluene.

Knowing that Eu^II^**1** can be formed *in situ* and that Zn^0^ does not promote the reductive coupling of benzyl chloride, we wanted to probe the catalytic activity of Eu^II^**1**. A benzyl chloride coupling reaction was performed starting from EuCl_3_ (10 mol%) and **1** (10 mol%). This reaction yielded 1,2-diphenylethane (80 ± 10%) and toluene (11 ± 2%) in six hours (Fig. 3B). The variation in yields is likely due to the heterogeneity of the reaction mixture and small differences in stir rate, causing a variability in light penetration. These experiments demonstrate that the photoredox reaction can be rendered catalytic (∼8 turnovers) in europium.

To determine how catalyst loading influenced product formation, the loading of EuCl_3_ and **1** were systematically varied, keeping ten equivalents of Zn^0^ relative to benzyl chloride constant, and yields were compared at six hours. Benzyl chloride coupling reactions were performed at catalyst loadings of 5, 1, and 0.5 mol%. Yields of 1,2-diphenylethane of 71 ± 5% (∼14 turnovers), 70 ± 5% (∼70 turnovers), and 60 ± 3% (∼120 turnovers), respectively, were observed. Toluene was also formed at yields of 12 ± 2, 21 ± 2, and 26 ± 1% for 5, 1, and 0.5% catalyst loadings, respectively. This trend demonstrates that decreased catalyst loading correlates to increased toluene production. At a much lower catalyst loading (0.005%), only toluene formation was observed. These results indicate that the precatalyst operates efficiently at low concentrations but is likely in competition with zinc for reduction *versus* reductive coupling.

After examining the catalytic utility of Eu^II^**1**, we were interested in examining the effect of water on the system because all of the reactions to this point were performed under anhydrous conditions. To introduce water into the system, EuCl_3_·6H_2_O was used as the Eu^III^ source and the samples were prepared in a wet glovebox (water allowed but no molecular oxygen). Reactions of the catalytic reductive coupling of benzyl chloride under these wet conditions were prepared at 10 mol% catalyst loading, and the formation of 1,2-diphenylethane in yields of 80 ± 3% was observed. These yields are not different from those of reactions performed under anhydrous conditions, indicating that small amounts of water have no significant effect on the performance of the precatalyst.

To determine if Eu^III^ remains complexed after the oxidation of Eu^II^, luminescence intensities were compared of solutions containing EuCl_3_, EuCl_3_ in the presence of **1**, and Eu^II^**1** that was opened to air to oxidize (Fig. S17†). The spectra were normalized to the ^5^D_0_ → ^7^F_1_ transition at 591 nm that is insensitive to ligand environment, and the emission intensities of the spectra were compared at the ^5^D_0_ → ^7^F_2_ transition (610–630 nm) that is hypersensitive to ligand environment.^26^ The change in spectral profile of the ^5^D_0_ → ^7^F_2_ transitions indicates that there is an interaction between Eu^III^ and **1**, but the exact nature of this interaction is ambiguous.

**Scheme 1:**
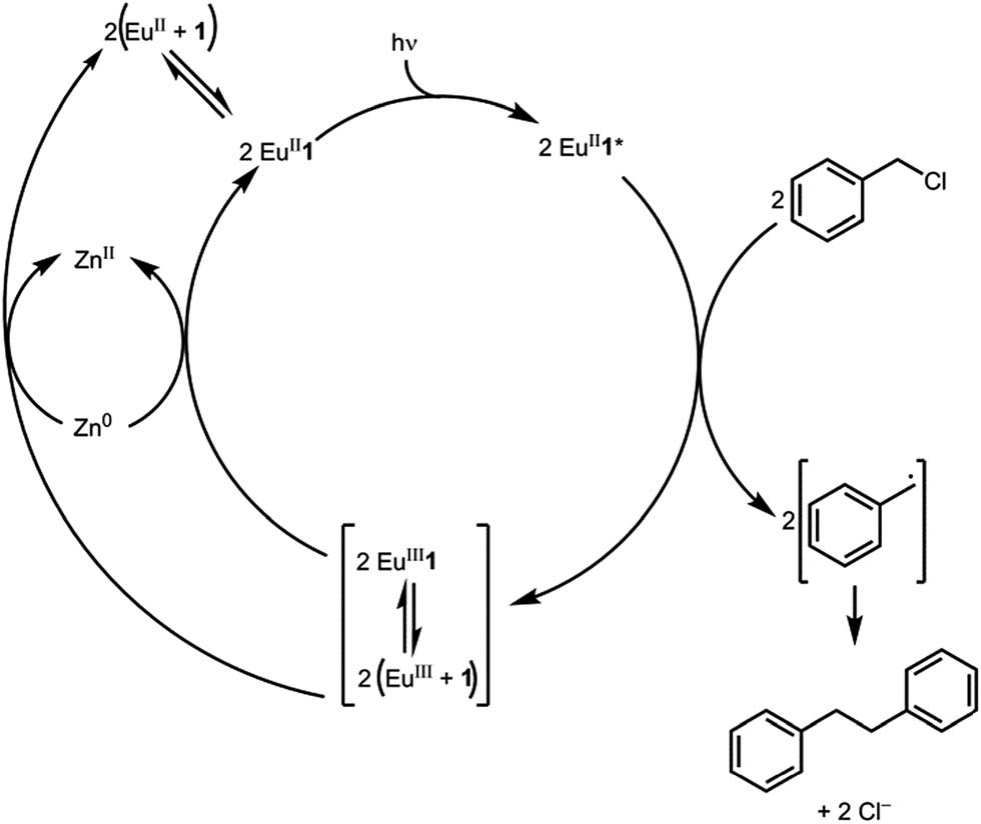
Proposed catalytic cycle.

Based on the data presented here, we propose that the photocatalytic reductive coupling of benzyl chloride using Eu^II^**1** proceeds through the catalytic cycle shown in Scheme 1. From luminescence experiments, Eu^II^**1** is excited by blue light into an excited state (Eu^II^1*). Two molecules of Eu^II^1* reduce two molecules of substrate through a collisional electron transfer based on Stern–Volmer analyses, followed by reductive coupling of substrate molecules. The electron transfer also generates Eu^III^ that interacts with **1** to some extent. Zn^0^ reduces Eu^III^ to Eu^II^ either as the complex or the uncomplexed ion. Spectroscopic evidence (Fig. S17†) supports the presence of interactions between Eu^III^ and **1**, but this evidence is not conclusive with respect to the nature of speciation of the trivalent ion. Regardless of the extent of encapsulation of Eu^III^ by **1**, reduction by Zn^0^ regenerates Eu^II^**1**, evidenced by spectroscopy and the crystal structure in Fig. 4, restarting the catalytic cycle.

## Conclusions

We have described the first report of photoredox catalysis based on europium. Exposure of Eu^II^**1** to visible light forms an excited state with a calculated electrochemical potential that rivals SmI_2_ in the presence of hexamethylphosphoramide, has a long luminescence lifetime, is tolerant of protic solvents and some H_2_O, and can be assembled *in situ* starting from air-stable and relatively inexpensive EuCl_3_·6H_2_O. We expect that the mechanistic insight provided here will open the door for the study of visible-light-promoted photoredox catalysis using Eu^II^**1** in reactions that require large negative electrochemical potentials between –0.9 and approximately –3 V *vs.* Ag/AgCl, including challenging systems like unactivated halides such as aryl bromides. Furthermore, studies from our laboratory have shown that ligand modifications to Eu^II^**1** can influence its spectroscopic properties,25*a* and these modifications are likely to impact excited-state redox properties. Studies exploring ligand modifications and the scope of reactivity of Eu^II^**1** are underway in our laboratory.

## Conflicts of interest

There are no conflicts to declare.

## Supplementary Material

Supplementary information

Crystal structure data
