# Supplementary material for: First use of a divalent lanthanide for visible-light-promoted photoredox catalysis
Source: Chem Sci. 2017 Dec 21;9(5):1273–8. doi: 10.1039/c7sc02479g (PMC5890796; doi:10.1039/c7sc02479g)
Supplement: Supplementary file 1 [file SC-009-C7SC02479G-s001.pdf]

Electronic Supplementary Material (ESI) for Chemical Science.
This journal is © The Royal Society of Chemistry 2017

First Use of a Divalent Lanthanide for Visible-Light-Promoted Photoredox Catalysis

Tyler C. Jenks,‡ Matthew D. Bailey,‡ Jessica L. Hovey, Shanilke Fernando, Gihan Basnayake, Michael E. Cross, Wen Li and Matthew J. Allen*

Department of Chemistry, Wayne State University, Detroit, MI 48202, United States

‡These authors contributed equally.

*E-mail: mallen@chem.wayne.edu

Page	Content
S1	Table of contents
S2	Experimental procedures
S5	GC–MS data
S16	Cyclic voltammetry
S19	Crystallographic data
S19	Calculation of quantum yield
S21	Stern–Volmer analysis
S24	Binding study of Eu^{III} and **1**
S26	UV–visible and luminescence spectra of zinc-reduced Eu^{II}**1**
S28	Reactivity of Eu^{II}**1** with methanol
S28	Lifetime of Eu^{II}**1**
S29	References

Experimental procedures

Commercially available chemicals were of reagent-grade purity or better and were used without further purification unless otherwise noted. DriSolv anhydrous methanol and liquid substrates were degassed under reduced pressure prior to being brought into gloveboxes. Non-degassed ACS-grade methanol was used for dilutions performed outside of gloveboxes. Water was purified by using a PURELAB Ultra Mk2 water purification system (ELGA). Azacryptand 1,4,7,10,13,16,21,24-octaazabicyclo[8.8.8]hexacosane (**1**) was prepared by following a published procedure.[1]

Photoreactors were assembled using strips of multicolored light emitting diodes (LEDs) purchased from Lighting Ever (SKU 4100053-US, http://www.lightingever.com/rgb-led-strip-non-waterproof-4100053-us-a.html, accessed on 08/26/2016). Each reactor consisted of approximately 47 LEDs (0.16 W per LED) resulting in a total of ~7.6 W of light for each reactor. Reactors were placed on top of stir plates and fans (Vornado Flippi V6 Personal Air Circulator) were used to cool the reactors.

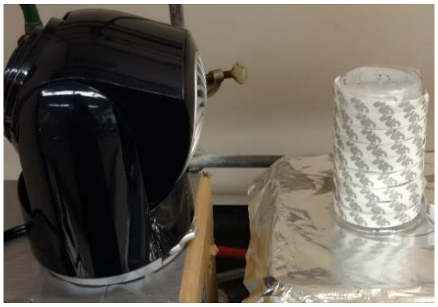

Figure S1 Picture of a photoreactor next to a cooling fan.

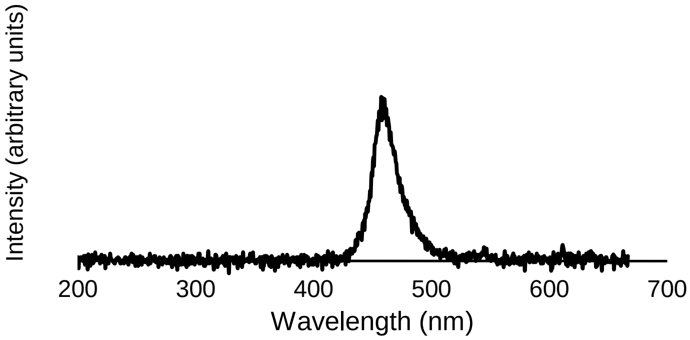

Figure S2 Emission profile of the blue light-emitting diodes used in the photoreactors showing a maximum emission band centered at 460 nm.

Stoichiometric reactions were prepared in a dry glovebox under an atmosphere of N_2. Samples of Eu^{II}**1** were prepared by mixing solid **1** (103.0–107.9 mg, 0.2779–0.2912 mmol, 1 equiv) with solid $EuCl_2$ (62.1–63.9 mg, 0.279–0.287 mmol, 1 equiv) and diluting with methanol to a total volume of 10.0 mL. The resulting solution was stirred for 2 h to yield a solution of Eu^{II}**1** (27.8–28.7 mM). Benzyl chloride solutions were prepared by diluting benzyl chloride (30.7–37.9 mg,

0.243–0.299 mmol, 1 equiv) with methanol to a total volume of 10.0 mL to make a benzyl chloride solution (24.3–29.9 mM). Reactions were prepared by adding the benzyl chloride solution (1.00 mL), the Eu^{II}**1** solution 1.00 mL), and methanol (1.00 mL) to a 20 mL vial equipped with a Teflon-coated stir bar. Vials were sealed with electrical tape, brought out of the glovebox, and placed in photoreactors to be illuminated with blue LEDs. For time progression studies, eight identical reaction vessels were prepared simultaneously from the same stock solutions. Starting at zero min (before irradiation), one reaction was stopped every five min for 35 min. After completion, each reaction mixture was removed from the photoreactor and diluted to 10.0 mL with ACS-grade methanol for analysis by gas chromatography–mass spectrometry (GC–MS) using method A (see GC–MS section below).

Substrate scope reactions were performed at stoichiometric catalyst concentrations as described in the stoichiometric reaction section and were prepared in a dry glovebox under an atmosphere of N_2. Eu^{II}**1** stock solutions were prepared as described in the stoichiometric reaction section. Substrate solutions were prepared by diluting the requisite amount of substrate (18.4–23.6 mg, 0.240–0.308 mmol, 1 equiv allyl chloride; 27.8–33.8 mg, 0.247–0.300 mmol, 1 equiv chlorobenzene; and 25.3–39.5 mg, 0.273–0.427 mmol, 1 equiv 2-chloro-2-methylpropane) with methanol to a total volume of 10.0 mL. Reactions were prepared by adding the respective substrate solution (1.00 mL), the Eu^{II}**1** solution (1.00 mL), and methanol (1.00 mL) to a 20 mL vial equipped with a Teflon-coated stir bar. Vials were sealed with electrical tape, brought out of the glovebox, and placed in photoreactors to be illuminated with blue LEDs for 30 min. Each reaction was extracted with GC–MS-grade *n*-pentane (5 × 3 mL) and the *n*-pentane extract was used for GC-MS analysis using method B for the allyl chloride and 2-chloro-2-methylpropane reactions and method C for the chlorobenzene reactions (see GC–MS section below).

Catalytic reactions (10% catalyst loading) starting from $EuCl_3$ were prepared in a dry glovebox, and catalytic reactions starting from $EuCl_3{\cdot}6H_2O$ were prepared in a wet glovebox. Both the dry and wet gloveboxes operate under an atmosphere of N_2. Separate stock solutions of **1** (101.2–105.7 mg, 0.2731–0.2852 mmol, 0.1 equiv), $EuCl_3$ (71.9–73.6 mg, 0.278–0.285 mmol, 0.1 equiv), and $EuCl_3{\cdot}6H_2O$ (103.1–113.0 mg, 0.2814–0.3084 mmol, 0.1 equiv) were prepared by dissolving the respective solid in methanol and diluting to 10.0 mL. Benzyl chloride stock solutions were prepared by diluting benzyl chloride (337.1–364.4 mg, 2.663–2.879 mmol, 1 equiv) with methanol to a total volume of 10.0 mL. Reactions were prepared by adding the benzyl chloride solution (1.00 mL), the respective Eu^{III} solution (1.00 mL), and the solution of **1** (1.00 mL) to a 20 mL vial containing approximately 176 mg of zinc dust (~10 equiv vs substrate) and a Teflon-coated stir bar. Reaction vessels were sealed with electrical tape before removal from the glovebox. Reactions were stirred for 2 h in the dark prior to being illuminated by blue LEDs. For time-progression studies, eight identical reaction vessels were prepared simultaneously from the same stock solutions. Starting at 0 h (after the 2 h stirring period but before irradiation), one reaction was stopped every hour for 7 h. After completion, each reaction mixture was removed from the photoreactor, filtered through Celite to remove zinc, and diluted to 100.0 mL with ACS-grade methanol for analysis by GC–MS using method A (see GC–MS section below).

Catalytic loading reactions (5, 1, and 0.5% catalyst loadings) were prepared in a dry glovebox under an atmosphere of N_2. Benzyl chloride stock solutions were prepared by diluting benzyl chloride (344.8–351.3 mg, 2.724–2.775 mmol, 1 equiv) with methanol to a total volume of 10.0 mL. Separate stock solutions of **1** (50.0–55.0 mg, 0.135–0.148 mmol, 0.05 equiv) and $EuCl_3$ (33.8–35.2 mg, 0.131–0.136 mmol, 0.05 equiv) were prepared by dissolving the respective solid in methanol and diluting to 10.0 mL. The 5% loading reactions received 1.00 mL of each stock solution; the 1% loading reactions received 1.00 mL of the benzyl chloride stock solution, 0.20 mL of the $EuCl_3$ and **1** stock solutions, and 1.60 mL of methanol; and the 0.5% loading reactions received 1.00 mL of the benzyl chloride stock solution, 0.10 mL of the $EuCl_3$ and **1** stock solutions, and 1.80 mL of methanol. Reactions were prepared in 20 mL vials containing approximately 176 mg of zinc dust (~10 equiv vs substrate) and a Teflon-coated stir bar, then sealed with electrical tape prior to removal from the glovebox. Reactions were stirred for 2 h in the dark prior to being illuminated by blue LEDs for 6 h. After completion, each reaction mixture was removed from the photoreactor, filtered through Celite to remove zinc, and diluted to 100.0 mL with ACS-grade methanol for analysis by GC–MS using method A (see GC–MS section below).

Control reactions were prepared in a dry glovebox under an atmosphere of N_2. The control reaction omitting light was prepared as described in the stoichiometric reaction section above, but the reaction vessel was wrapped in foil inside the photoreactor and stirred for 30 min. For the control reaction omitting europium, solid **1** (11.1 mg, 0.0300 mmol, 1 equiv) was weighed into a 20 mL vial with a Teflon-coated stir bar along with a solution of benzyl chloride (27 mM, 1.00 mL) and methanol (2.00 mL), and the reaction was stirred under irradiation from blue LEDs for 30 min. For the control reaction omitting **1**, solid $EuCl_2$ (14.4 mg, 0.0646 mmol, 2 equiv) was weighed into a 20 mL vial with a Teflon-coated stir bar along with a solution of benzyl chloride (27 mM, 1.00 mL) and methanol (2.00 mL), and the reaction was stirred under irradiation from blue LEDs for 30 min. All control reactions were sealed with electrical tape before being removed from the glovebox. After completion, each reaction mixture was removed from the photoreactor and diluted to 10.0 mL with ACS-grade methanol for analysis by GC–MS using method A (see GC–MS section below).

For the control reaction showing that zinc alone does not promote the coupling of benzyl chloride, zinc dust (~176 mg, 10 equiv) was weighed into a 20 mL vial with a Teflon-coated stir bar. To this vial was added a solution of benzyl chloride (114 mM, 3.00 mL). For the control showing the reaction of benzyl chloride with light, benzyl chloride (35.9–36.1 mg, 0.2863–0.285 mmol) was weighed into a vial containing methanol (3.00 mL). The reactions were stirred for 2 h in the dark before being exposed to blue LEDs for 6 h. All control reactions were sealed with electrical tape before being removed from the glovebox. After completion, each reaction mixture was removed from the photoreactor and diluted to 100.0 mL with ACS-grade methanol for analysis by GC–MS using method A (see GC–MS section below). The reactions containing zinc were filtered through Celite prior to dilution.

Gas chromatography–mass spectrometry (GC–MS) analyses were performed on a Shimadzu GC 2010 Plus gas chromatograph with a Shimadzu SH-Rxi-5Sil MS 30 m column and Shimadzu GCMS-QP2010 SE mass spectrometer with an electron impact ionization source. All GC methods used an injection temperature of 250 °C and split injection mode with a 5.0 split ratio. All MS methods used an ion source temperature of 200 °C, an interface temperature of 300 °C, an event time of 0.10 s, and a scan speed of 20,000 amu s^{-1}. Three separate methods (A, B, and C) were prepared to analyze the reactions and their specifications are listed below:

A. The temperature gradient for the GC method for the benzyl chloride coupling reaction starts at 50 °C and holds for 2.5 min, ramps to 300 °C at 50 °C per min, and holds at 300 °C for 1 min. MS acquisition time was set from 2.5 to 8.5 min. Calibration solutions for the starting materials [benzyl chloride (2.394–0.5984 mM)] and potential products [toluene (0.4027–0.1007 mM); 1,2-diphenylethane (1.569–0.3923 mM)] for the benzyl chloride reactions were prepared from commercially available materials in ACS-grade methanol. Integrated peak areas from the chromatograms were used to prepare standard curves and calculate yields of reactions.
B. The temperature for the GC method for the allyl chloride and 2-chloro-2-methylpropane coupling reactions holds at 26 °C for 6 min. MS acquisition time was set from 1.95 to 6 min. Calibration solutions for the starting materials [2-chloro-2-methylpropane (1.997–0.09987 mM)] and potential products [1,5-hexadiene (1.0229–0.05115 mM); 2,2,3,3-tetramethylbutane (1.0225–0.05113 mM)] of the allyl chloride and 2-chloro-2-methylpropane reactions were prepared in GC–MS-grade *n*-pentane. Allyl chloride did not yield a linear GC–MS response vs concentration; therefore, its calibration curve was not used. Integrated peak areas from the chromatograms were used to prepare standard curves and calculate yields of reactions.
C. The temperature gradient for the GC method for the chlorobenzene coupling reaction starts at 30 °C and holds for 5.5 min, ramps to 250 °C at 80 °C per min, and holds at 250 °C for 2 min. MS acquisition time was set from 2.05 to 8.69 min. Calibration solutions for the starting materials [chlorobenzene (2.213–0.1107 mM)] and potential products [benzene (1.061–0.05307 mM); biphenyl (1.010–0.05049 mM)] of the chlorobenzene reactions were prepared in GC–MS-grade *n*-pentane. Integrated peak areas from the chromatograms were used to prepare standard curves and calculate yields of reactions.

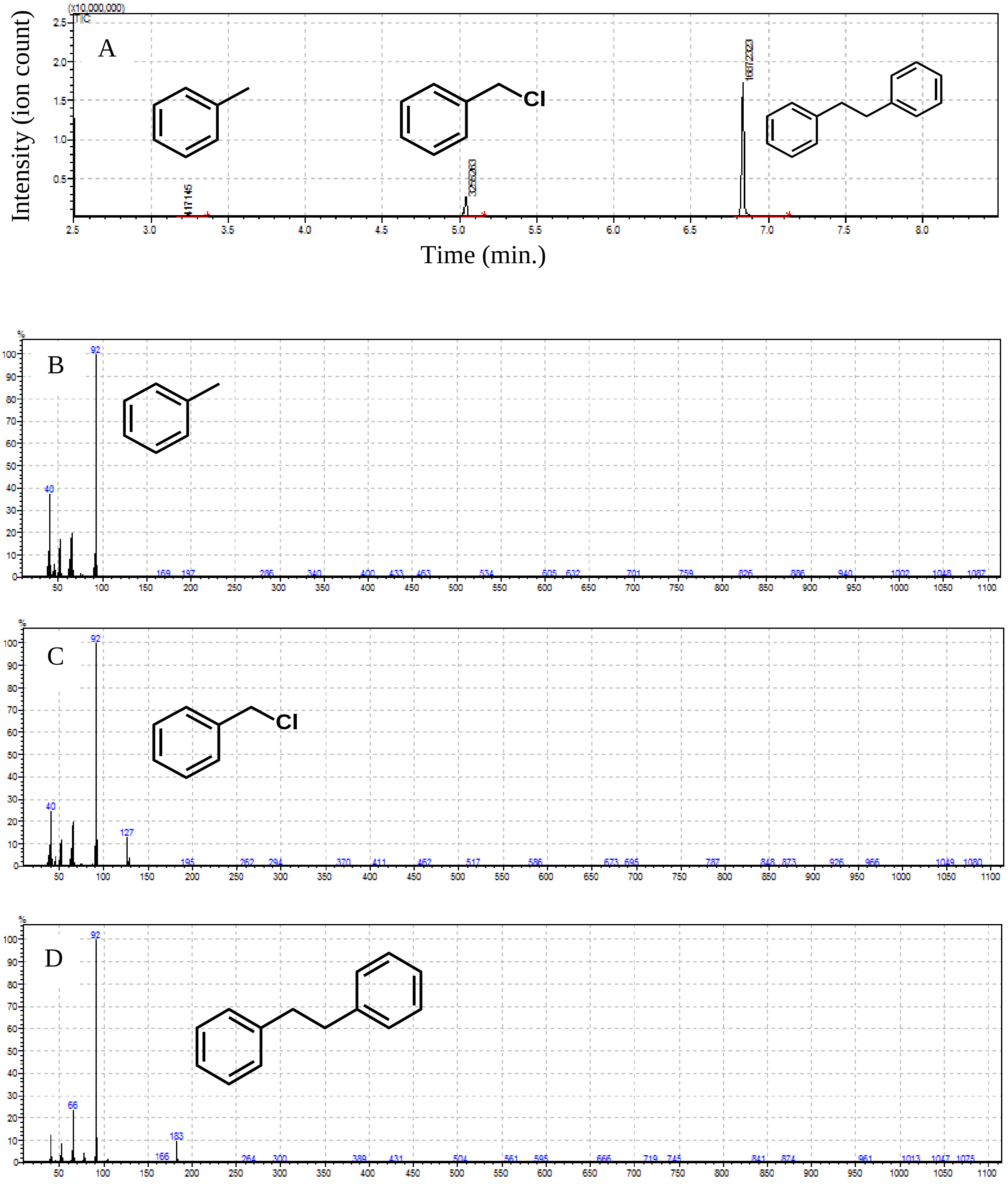

Figure S3 A. Representative GC–MS chromatogram of a stoichiometric benzyl chloride coupling reaction. B. Mass spectrum of the peak at 3.25 min corresponding to toluene. C. Mass spectrum of the peak at 5.05 min corresponding to benzyl chloride. D. Mass spectrum of the peak at 6.85 min corresponding to 1,2-diphenylethane.

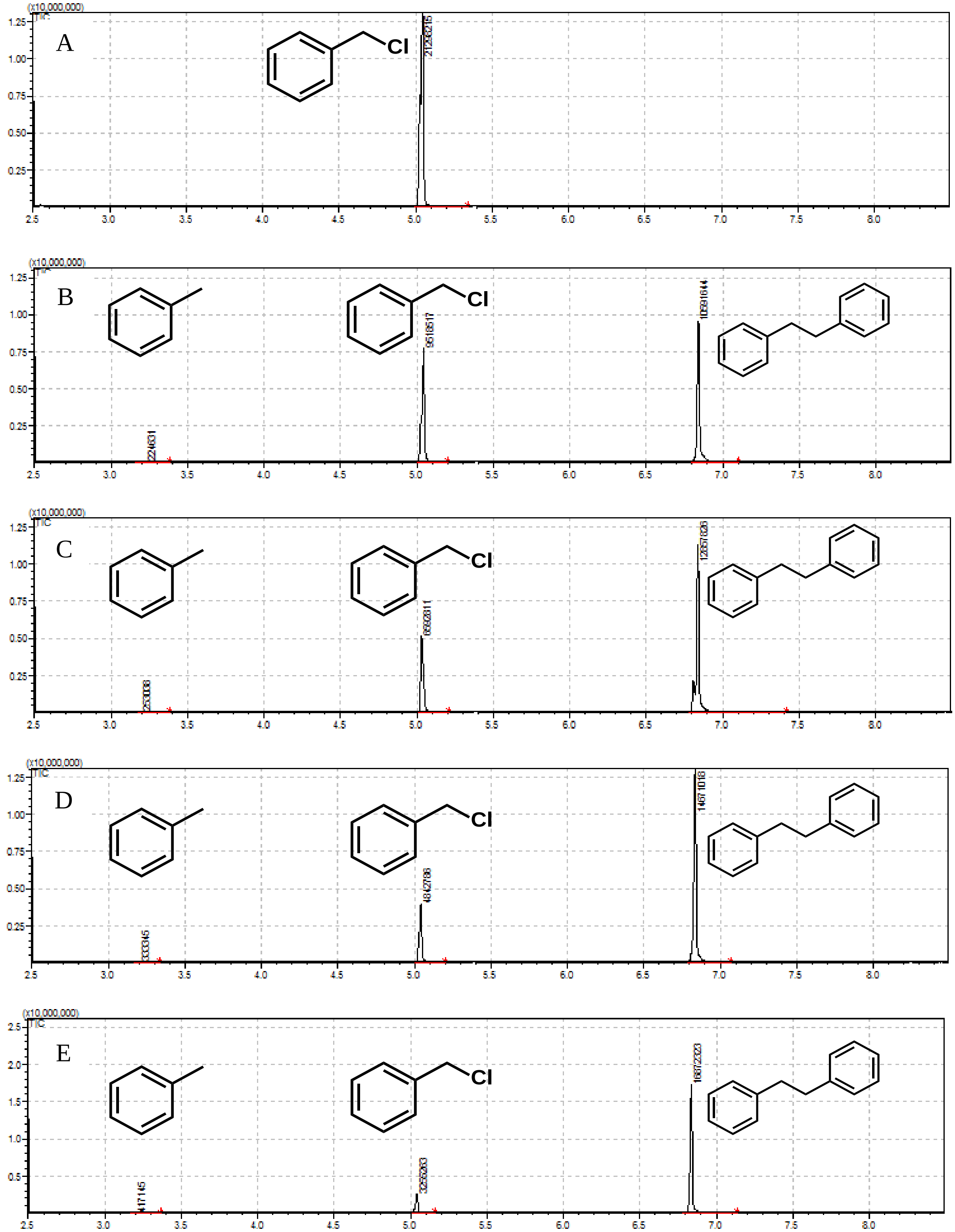

Figure S4 Part 1. Full caption can be found on the following page.

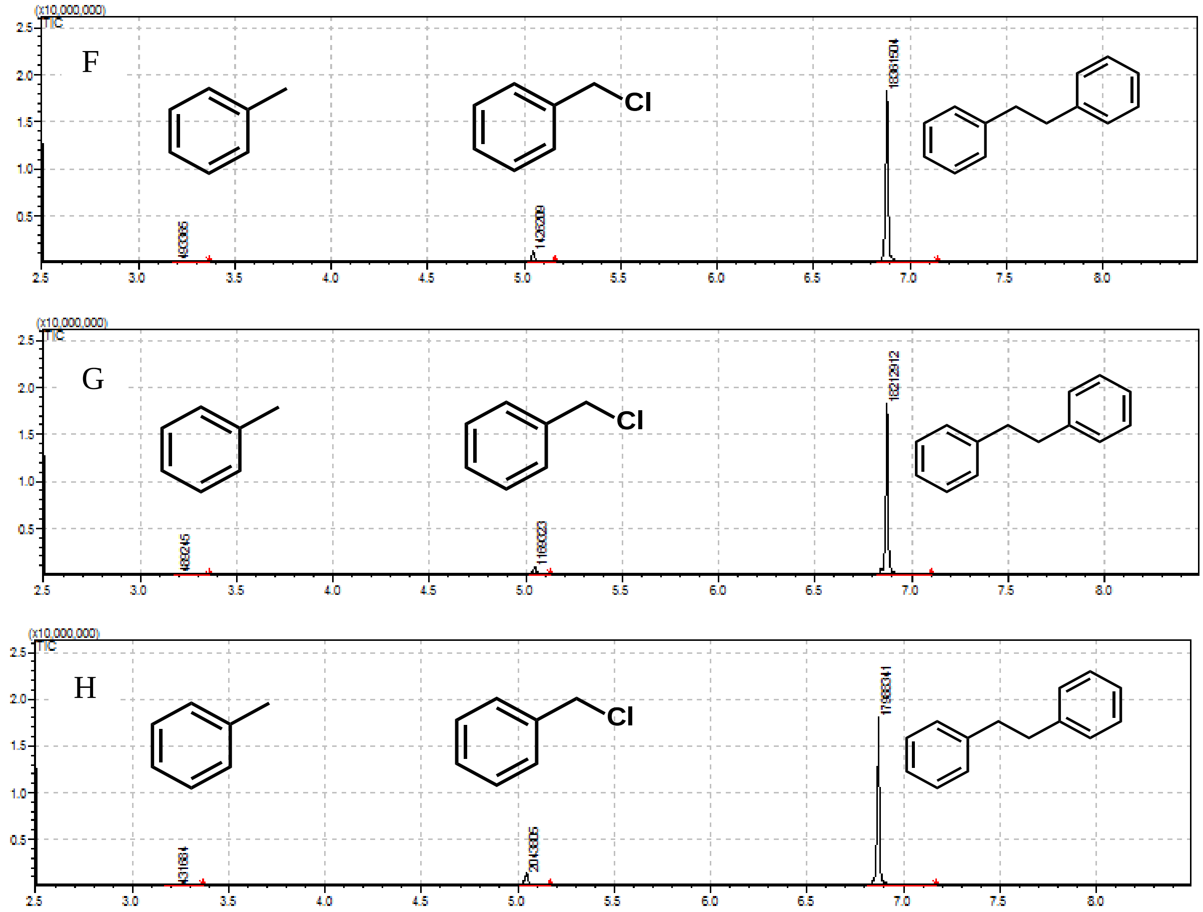

Figure S4 Continued. Representative GC–MS chromatograms of a time progression of stoichiometric benzyl chloride coupling reaction. Each chromatogram is of a separate reaction stopped after A. 0, B. 5, C. 10, D. 15, E. 20, F. 25, G. 30, and H. 35 min.

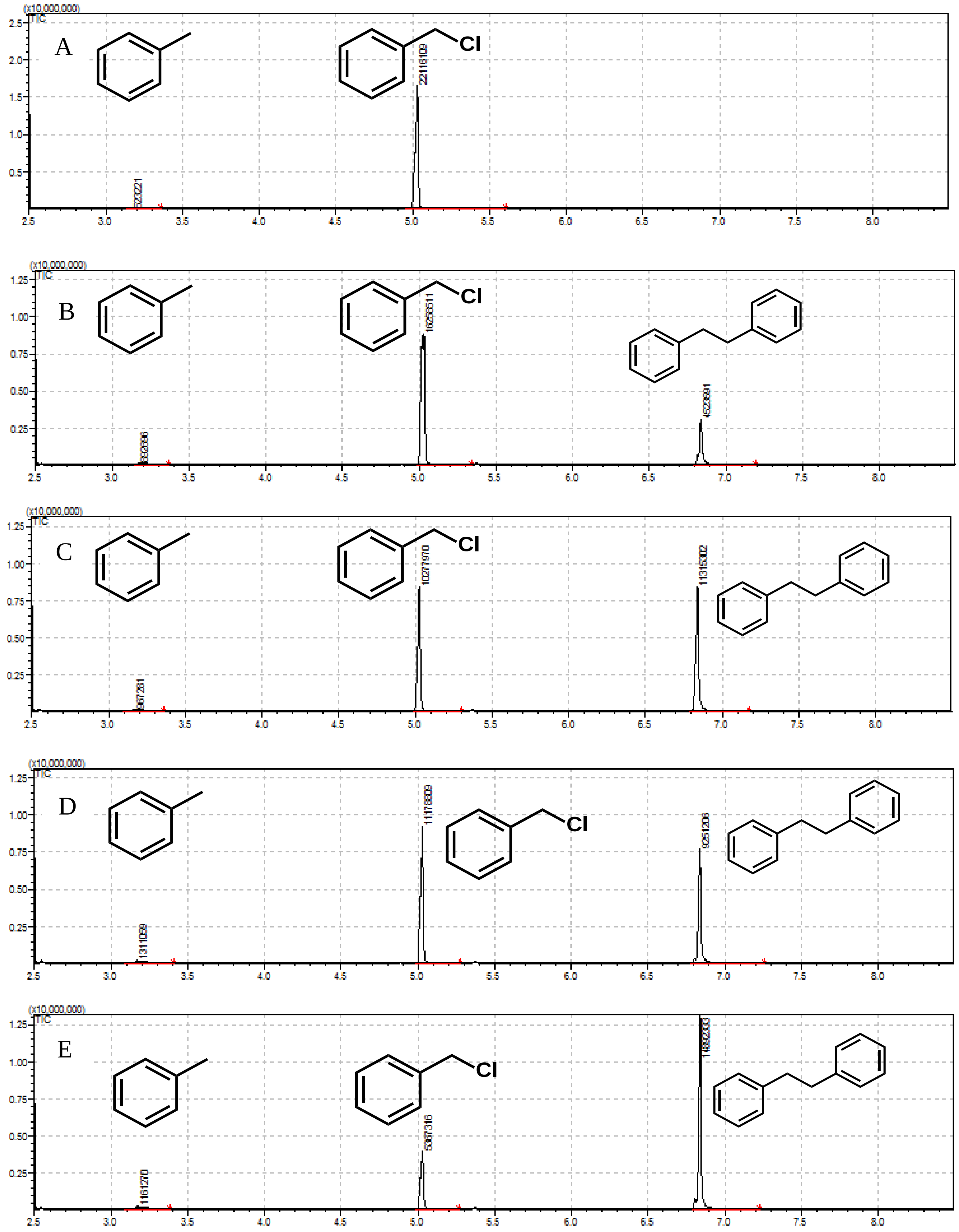

Figure S5 Part 1. Full caption can be found on the following page.

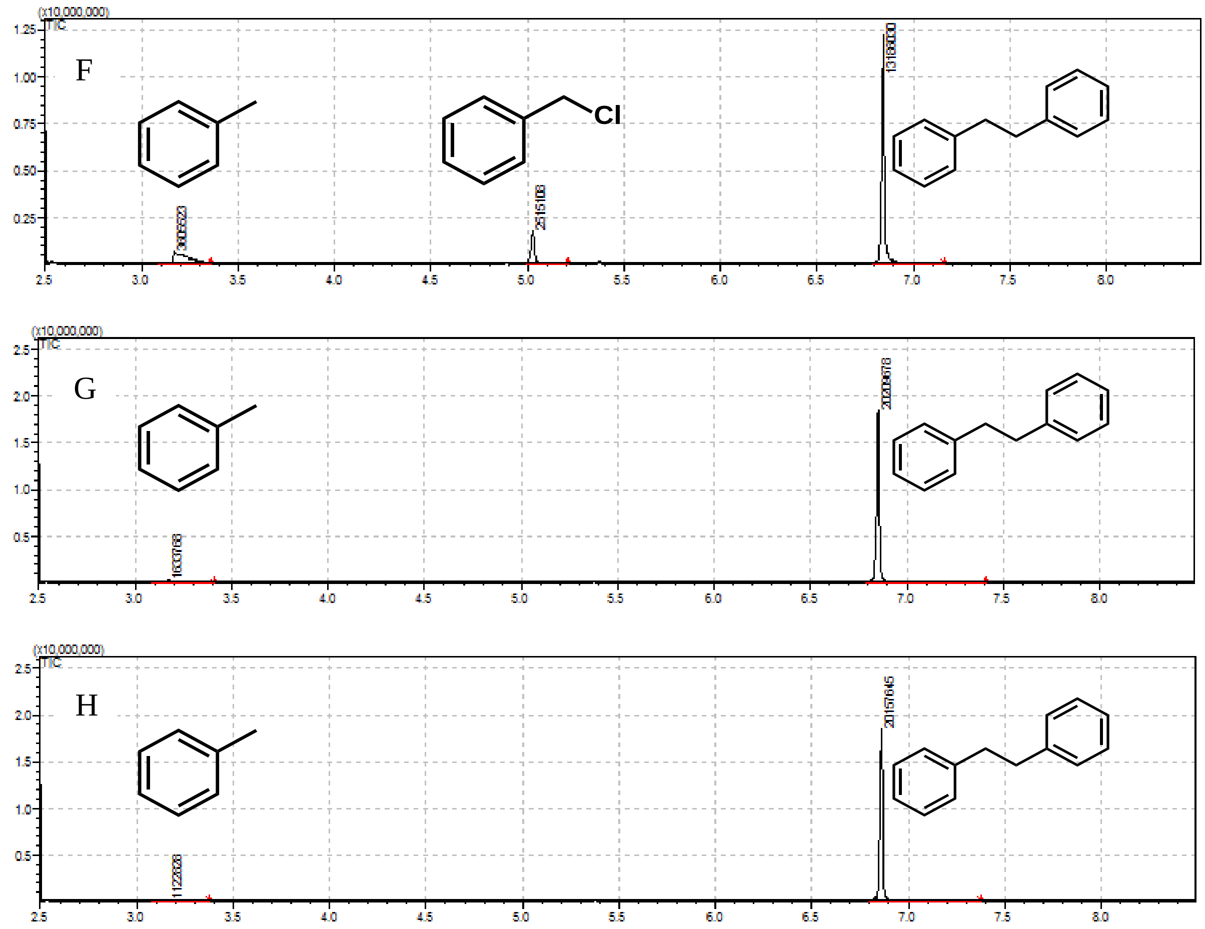

Figure S5 Continued. Representative GC–MS chromatograms of a time progression of catalytic benzyl chloride coupling reactions. Each chromatogram is of a separate reaction stopped after A. 0, B. 1, C. 2, D. 3, E. 4, F. 5, G. 6, and H. 7 h.

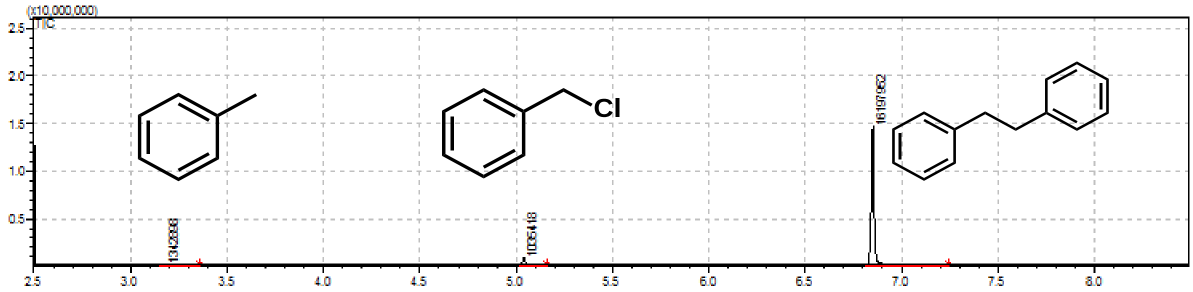

Figure S6 Representative GC–MS chromatogram of a catalytic benzyl chloride coupling reaction prepared in a wet glovebox starting from $EuCl_3 \cdot 6H_2O$.

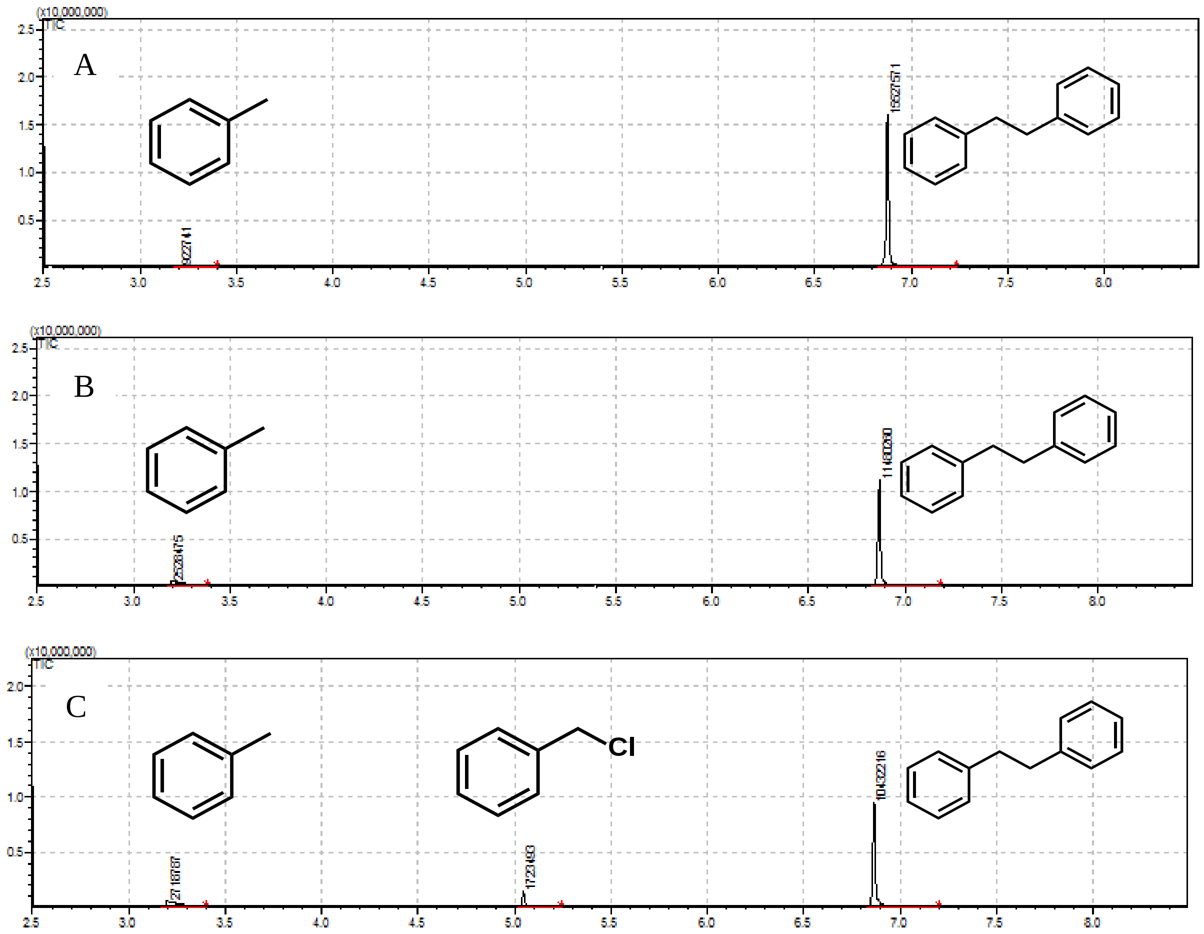

Figure S7 Representative GC–MS chromatograms of benzyl chloride coupling reactions run at catalyst loadings of A. 5, B. 1, and C. 0.5%.

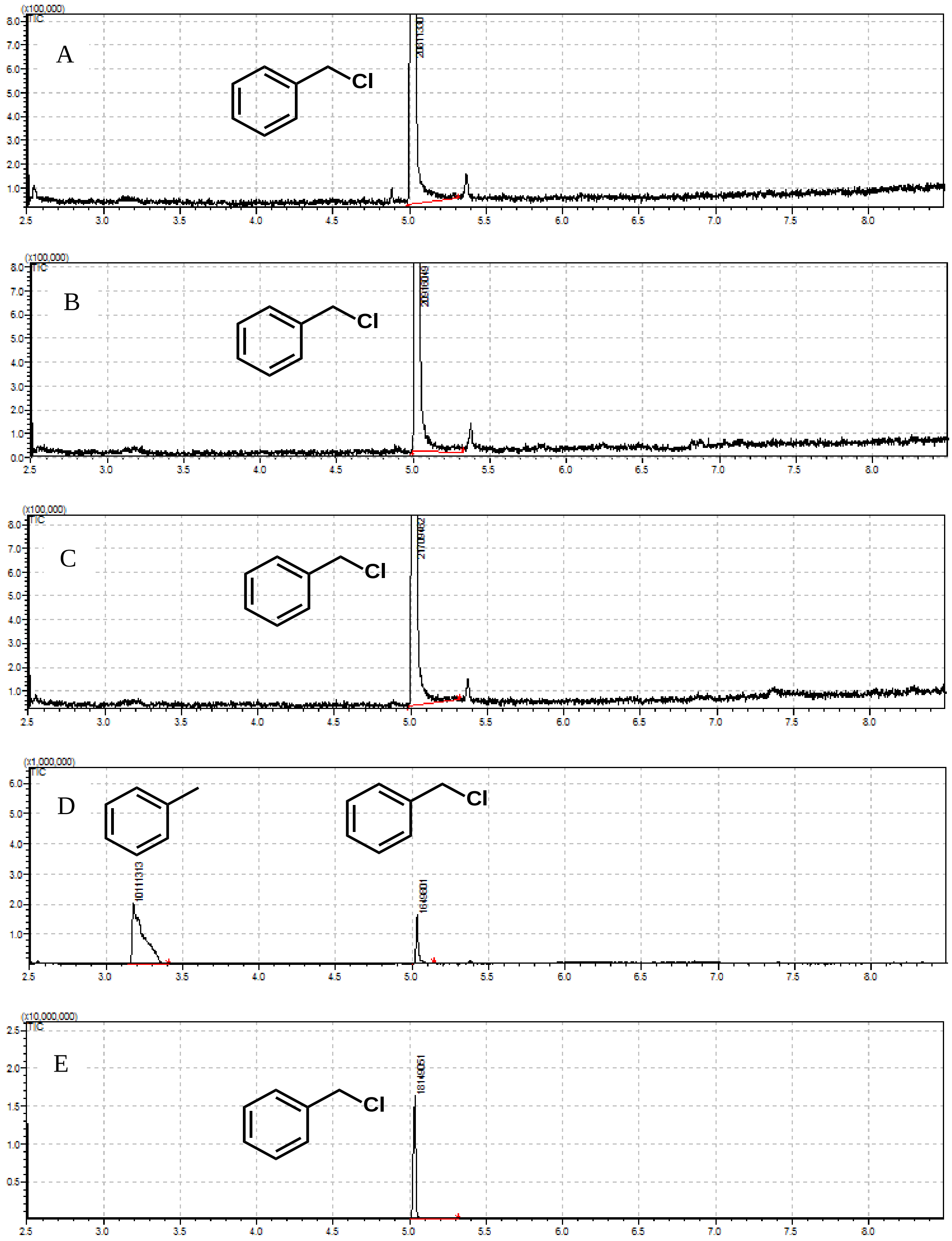

Figure S8 GC–MS chromatograms of control reactions A. in the absence of light; B. without europium; C. without **1**; D. with only zinc and benzyl chloride; and E. with only benzyl chloride in solution.

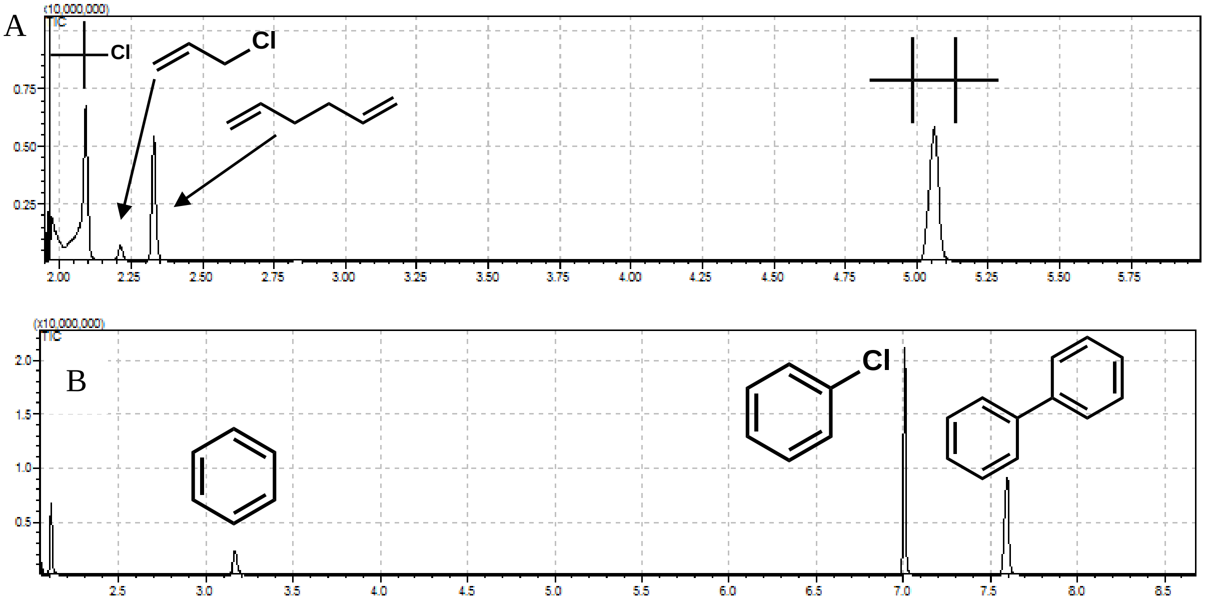

Figure S9 Representative GC–MS chromatograms of the calibration solutions for A. allyl chloride and 2-chloro-2-methylpropane coupling reactions and B. the chlorobenzene coupling reaction.

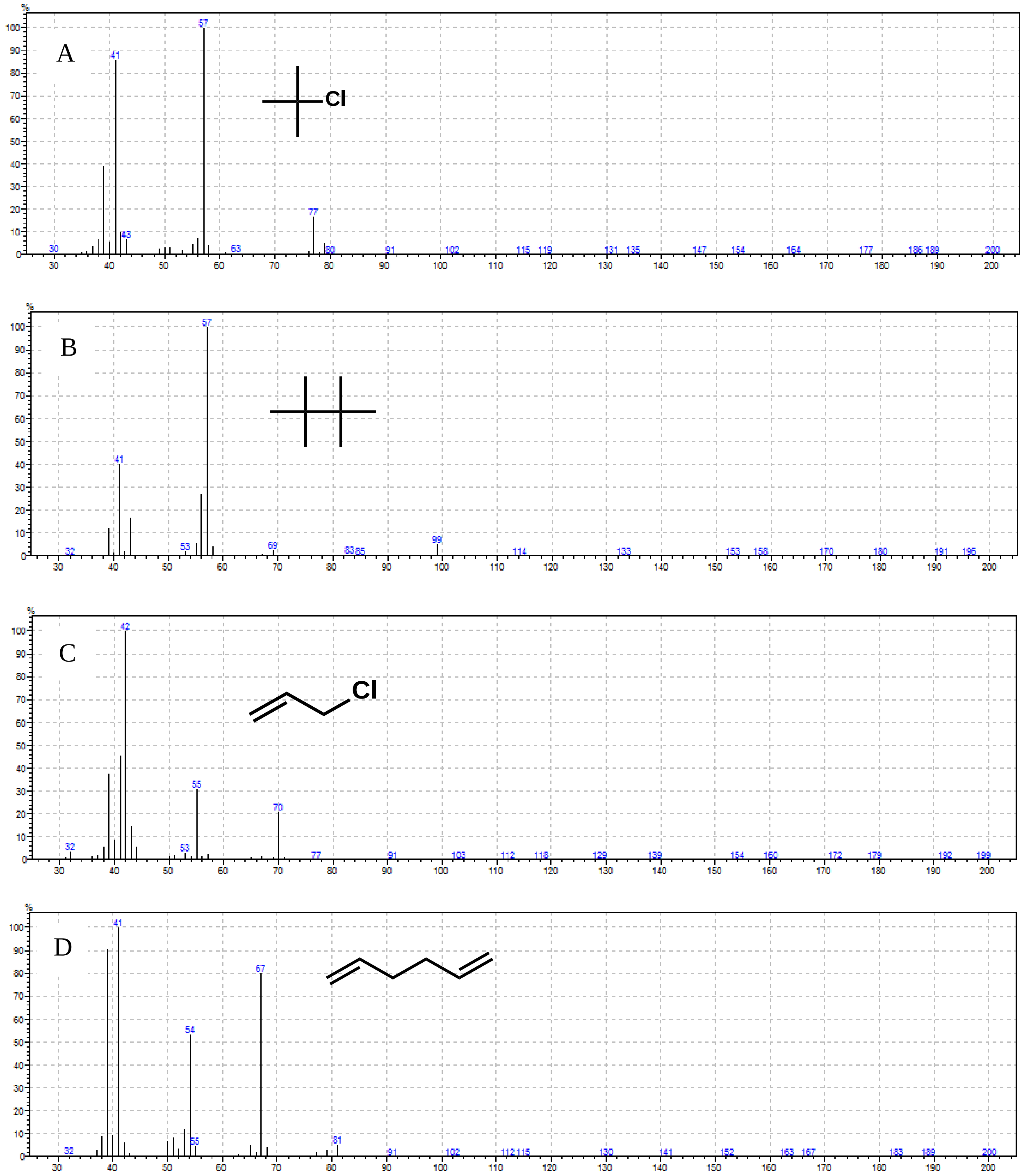

Figure S10 Part 1. Complete caption can be found on the following page.

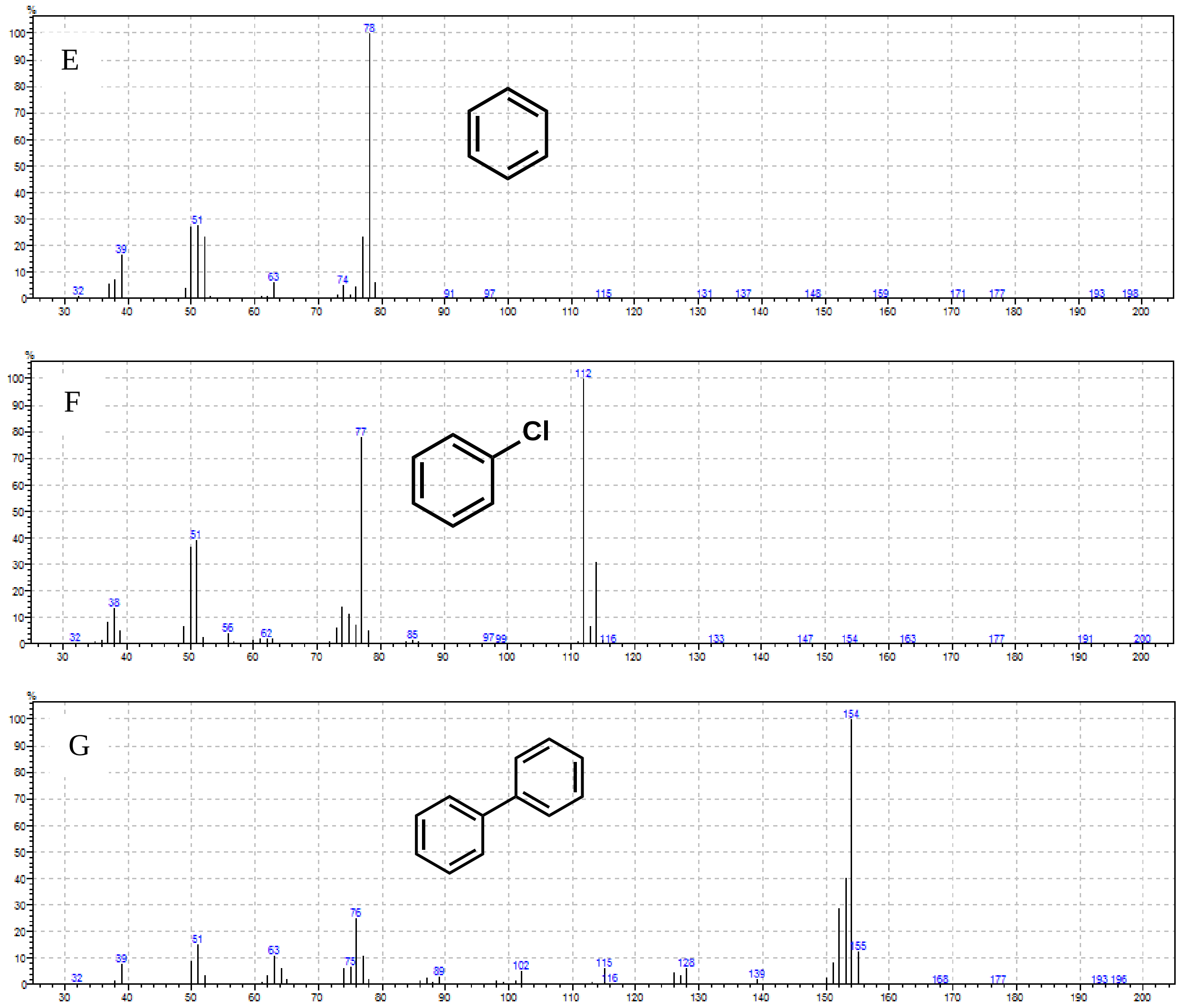

Figure S10 Continued. Mass spectra of A. 2-chloro-2-methylpropane, B. 2,2,3,3-tetramethylbutane, C. allyl chloride, D. 1,5-hexadiene, E. benzene, F. chlorobenzene, and G. biphenyl.

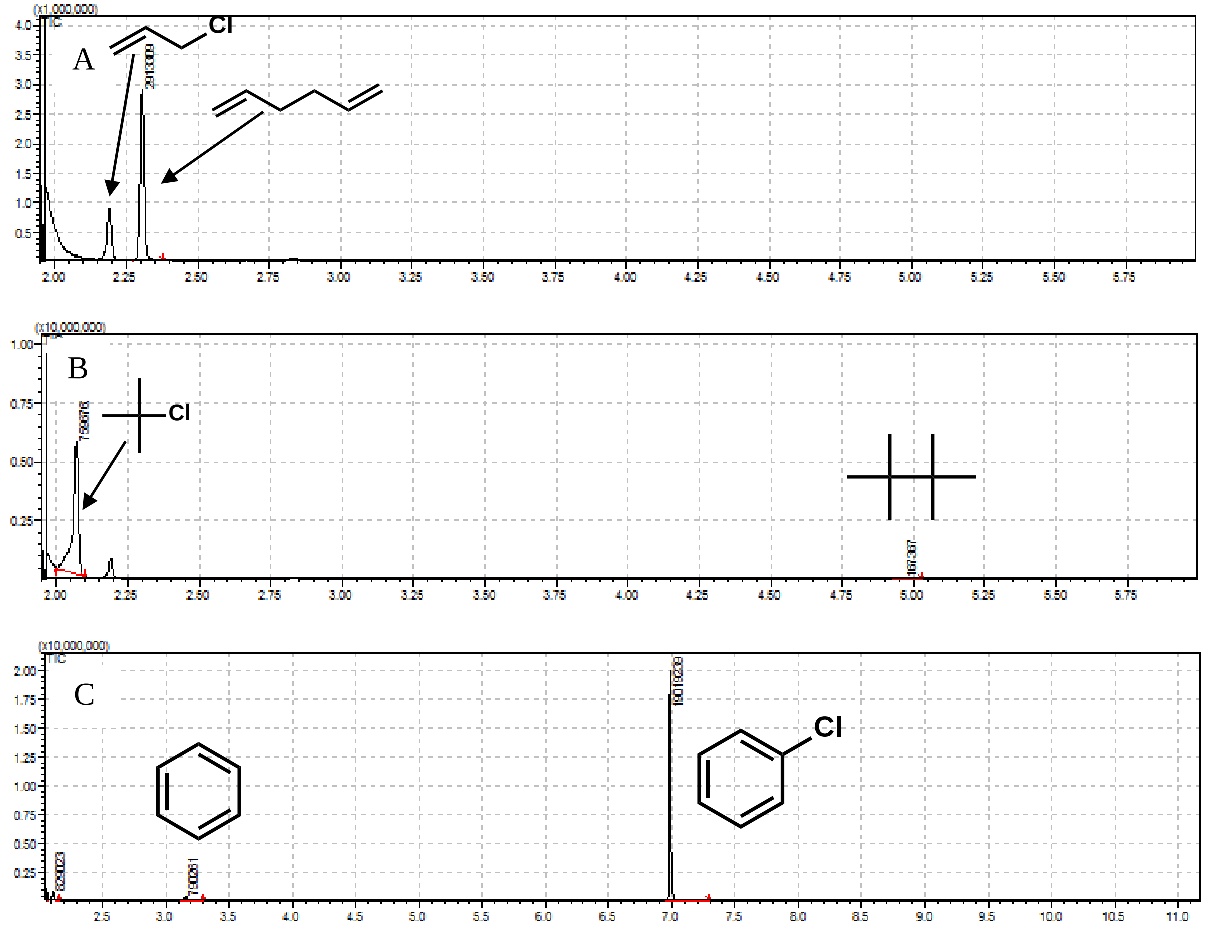

Figure S11 Representative GC–MS chromatograms of the products of the A. allyl chloride coupling reaction, B. 2-chloro-2-methylpropane coupling reaction, and C. chlorobenzene coupling reaction.

Cyclic voltammetry (CV) was performed on a Pine Wavenow USB potentiostat under an atmosphere of N_2 with a glassy carbon working electrode, a platinum wire counter electrode, and a Ag/AgCl reference electrode. All potentials are reported relative to Ag/AgCl. For the CV of Eu^{II}**1**, a solution of $Eu(OTf)_3$ (4.97 mM), **1** (28.7 mM), and tetraethylammonium perchlorate (48.1 mM) in anhydrous *N*,*N*-dimethylformamide (DMF) was used for the analysis, and the potential was found to be –0.90 V. Acquisition parameters were eight segments, an initial potential of –1.5 V (rising), an upper potential of 0 V, a lower potential of –1.5 V, and a sweep rate of 100 mV s^{-1}. For the CVs of the substrates, solutions of the substrates (90 mM) were prepared in anhydrous DMF with tetraethylammonium perchlorate (5 equiv) as the supporting electrolyte. Each acquisition consisted of eight scans with a sweep rate of 100 mV s^{-1}. Acquisition parameters and cathodic potentials for each substrate are as follows:

Benzyl chloride: The cathodic potential was found to be –2.34 V vs Ag/AgCl. Acquisition parameters were an initial potential of –2.75 V (rising), an upper potential of 0 V, and a lower potential of –2.75 V.

Allyl chloride: The cathodic potential was found to be –2.35 V vs Ag/AgCl. Acquisition parameters were an initial potential of –2.75 V (rising), an upper potential of 0 V, and a lower potential of –2.75 V.

Chlorobenzene: The cathodic potential was found to be –2.93 V vs Ag/AgCl. Acquisition parameters were an initial potential of –3.25 V (rising), an upper potential of 0 V, and a lower potential of –3.25 V.

2-chloro-2-methylpropane: The cathodic potential was found to be –3.05 V vs Ag/AgCl. Acquisition parameters were an initial potential of –3.25 V (rising), an upper potential of 0 V, and a lower potential of –3.25 V.

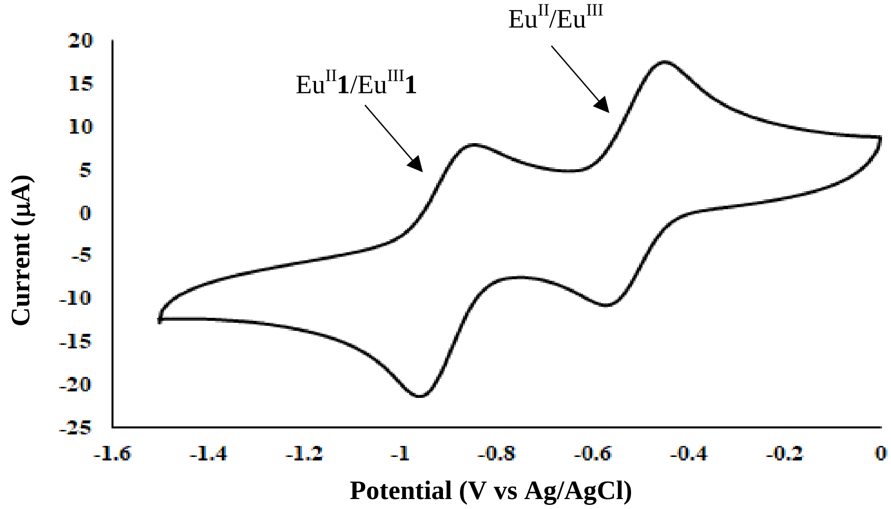

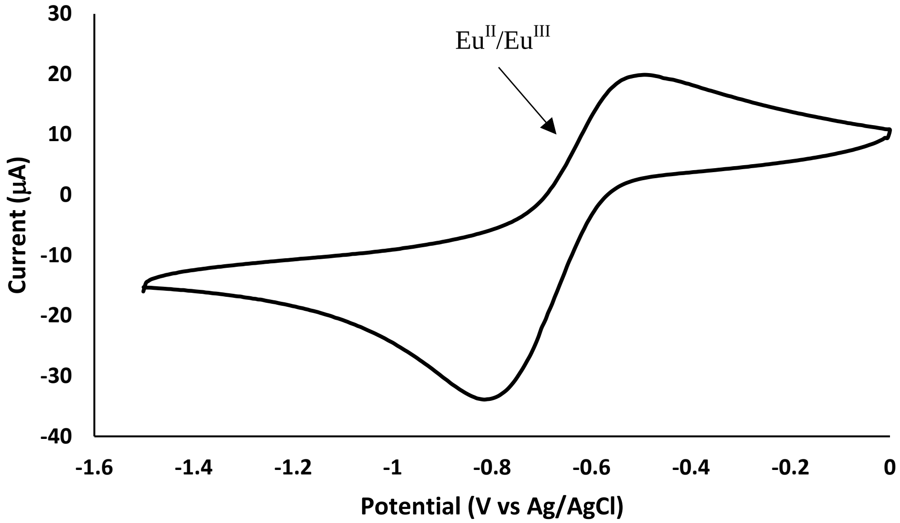

Fig. S12 Top: Cyclic voltammogram of $Eu(OTf)_3$ and **1** in DMF. Bottom: Cyclic voltammogram of $Eu(OTf)_3$ in DMF. Upon addition of ligand **1** to a solution of $Eu(OTf)_3$, a new peak arises with an $E_{1/2}$ of –0.9 V vs Ag/AgCl.

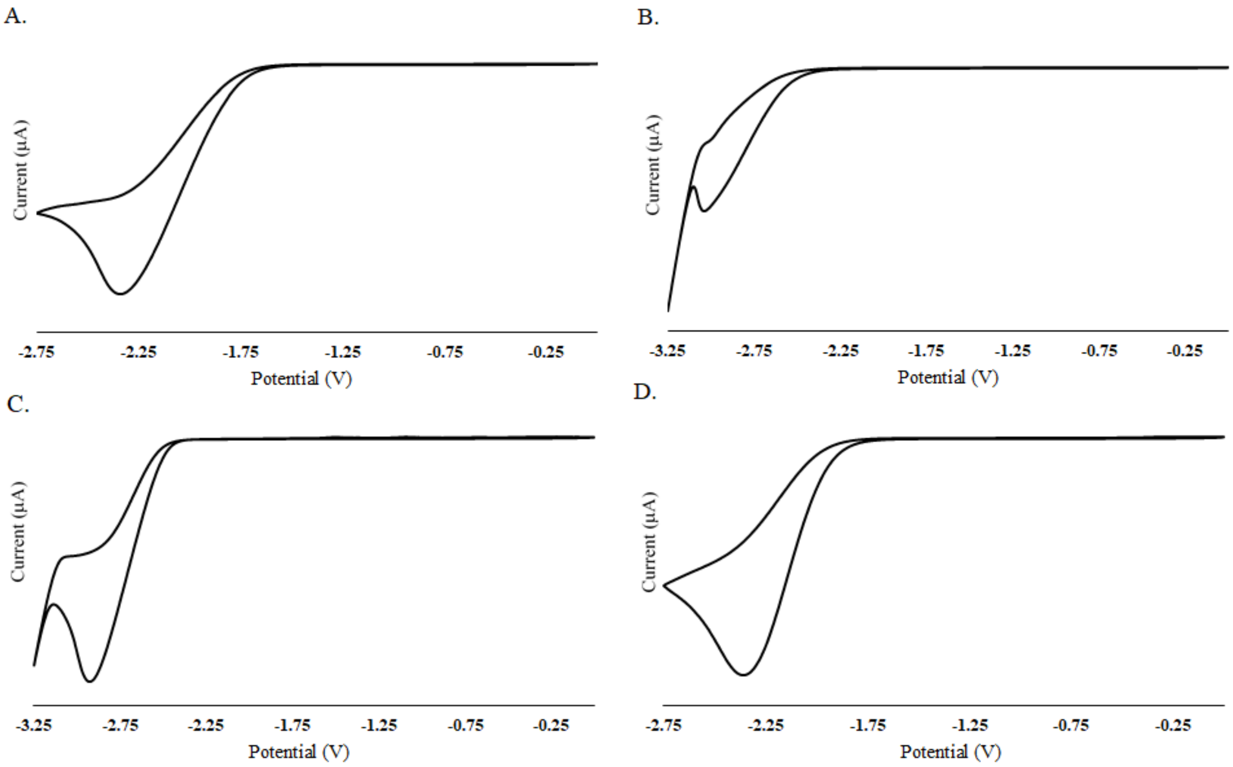

Fig. S13 Cyclic voltammograms of A. benzyl chloride, B. 2-chloro-2-methylpropane, C. chlorobenzene, and D. allyl chloride. All potentials are relative to Ag/AgCl.

Crystallographic data

Data were collected on a Bruker Apex-II Kappa geometry diffractometer using Mo Kα radiation. Spectral collection was performed with a charge coupled device and the temperature of the crystal was maintained at 100 K using an Oxford Cryostream low-temperature device. An initial solution was found using the method of intrinsic phasing via ShelXT[2] and further refined by the method of least squares using ShelXL[3] interfaced with OLEX2 and ShelXle.[4]

The Eu^{II}-containing complex crystallized in the space group *Pbca* with one cationic unit of $[Eu\mathbf{1}]^{2+}$, one anionic unit of $[ZnCl_4]^{2-}$, and one molecule of methanol in the asymmetric unit. All non-hydrogen atoms were refined anisotropically. The ethylene unit of C15 and C16 was found to have two positions, one with approximately 80% occupancy and the other with approximately 20% occupancy. The crystallographic data can be found in the Cambridge structural database under CCDC #1539923. These data are provided free of charge by the Cambridge Crystallographic Data Centre.

Table S1 Crystallographic properties of $[Eu^{II}\mathbf{1}][ZnCl_4]$:

Chemical Formula	$C_{19}H_{46}Cl_4EuN_8OZn$
Formula Weight	761.77
Temperature	100 K
Wavelength	0.71073 Å
Crystal System	orthorhombic
Space Group	*Pbca*
Unit Cell Dimensions	a = 16.9858(10) Å
	b = 18.4496(10) Å
	c = 18.8816(11) Å
	$\alpha = 90^{o}$
	$\beta = 90^{o}$
	$\gamma = 90^{o}$
Volume	5917.1(6) $Å^3$
Z	8
Density (calculated)	1.707 g cm^{-3}
Absorption Coefficient	3.300 mm^{-1}
F(000)	3080.0

Determination of quantum yield

The quantum yield of $Eu^{II}\mathbf{1}$ was determined using a comparative method with sodium fluorescein and coumarin-153 as standards and an excitation wavelength of 460 nm. Absorbance spectroscopy from 200 to 800 nm (with 5 nm resolution) was taken using a Varian Cary 50 Bio UV–visible spectrophotometer to obtain absorbance values at 460 nm. For each set of samples, a corresponding solvent blank was used to zero the instrument before each acquisition. Fluorometry was performed on a Jobin Yvon Fluoromax-4P spectrophotometer using λ_{ex} of 460 with a 1 nm slit width, and λ_{em} collected from 470 to 800 nm with a 1 nm slit width at 1 nm resolution for each solution. Luminescence data was integrated over the full domain for each

sample. Each integrated emission intensity was plotted against its corresponding absorbance at 460 nm.

Stock solutions of sodium fluorescein in $NaOH_{aq}$ (0.1 M) and coumarin-153 in ethanol were prepared and diluted until absorbance at 460 nm was ~0.13 for each solution. Standard values for sodium fluorescein (0.91) and coumarin-153 (0.52) were obtained from a published source.[5]

Coumarin-153 was first measured as an undiluted stock solution. Each subsequent coumarin-153 solution was generated by removal of 0.8 mL of solution from the previous sample followed by addition of 0.8 mL of methanol. Samples were agitated by hand before each measurement.

Sodium fluorescein was measured first as an undiluted stock solution. Each subsequent sodium fluorescein solution was generated by removal of 0.8 mL of solution from the previous sample followed by addition of 0.8 mL of methanol. Samples were agitated by hand before each measurement.

Each standard was evaluated as an unknown versus the other standard using Equation 1.[6] Refractive indices were obtained from the CRC Handbook.[7]

Equation 1: $$\phi_u = \phi_{st}\left(\frac{Grad_u}{Grad_{st}}\right)\left(\frac{\eta_u^2}{\eta_{st}^2}\right)$$

Coumarin-153 from sodium fluorescein:

ϕ_{st} = 0.91, $Grad_{st}$ = 71.418, η_{st} = 1.3344, $Grad_u$ = 37.154, η_u = 1.3611

ϕ_u = 0.48 in ethanol

Sodium fluorescein from coumarin-153:

ϕ_{st} = 0.52, $Grad_{st}$ = 37.154, η_{st} = 1.3611, $Grad_u$ = 71.418, η_u = 1.3344

ϕ_u = 0.98 in 0.1 M $NaOH_{aq}$

Solutions of Eu^{II}**1** as well as a methanol blank were prepared and sealed with electrical tape in a nitrogen glovebox in accordance with Table S2. Samples were brought out of the glovebox and immediately measured for absorbance and integrated emission using the same parameters as used with both standards.

Table S2 Quantum yield cuvette preparation.

Sample	Eu**1**Cl_2, 3 mM (mL)	Methanol (mL)	Total volume (mL)
1	0	3	3
2	0.2	2.8	3
3	0.4	2.6	3
4	0.6	2.4	3

Eu^{II}**1** from sodium fluorescein:

ϕ_{st} = 0.91, $Grad_{st}$ = 71.418, η_{st} = 1.3344, $Grad_u$ = 28.129, η_u = 1.3288

ϕ_u = 0.357 in methanol

Eu^{II}**1** from coumarin-153:

ϕ_{st} = 0.52, $Grad_{st}$ = 37.154, η_{st} = 1.3661, $Grad_u$ = 28.129, η_u = 1.3288

ϕ_u = 0.384 in methanol

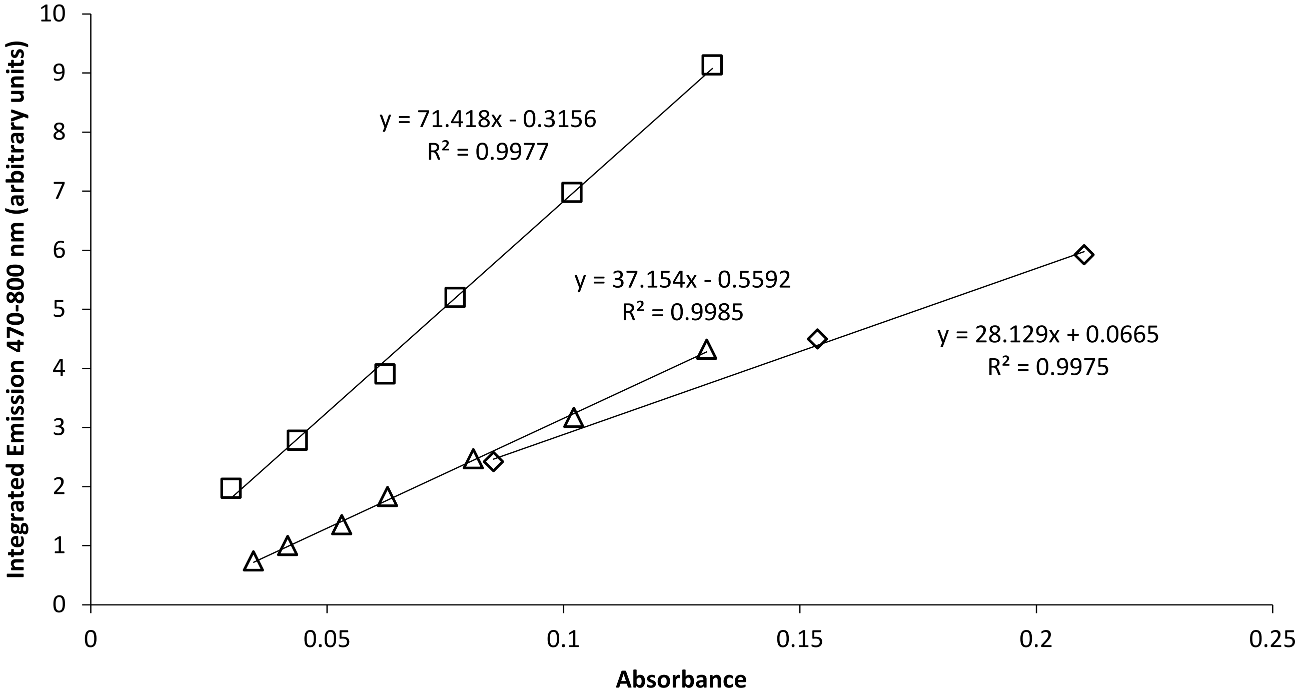

Figure S14 Integrated emission (λ_{ex} = 460, integrated from 470–800 nm) versus Absorbance (λ = 460 nm) for sodium fluorescein in 0.1 M $NaOH_{aq}$ (squares), coumarin-153 in ethanol (triangles), and Eu^{II}**1** in methanol (diamonds).

Ambient-temperature Stern–Volmer analyses

Solutions of Eu^{II}**1**, benzyl chloride, and allyl chloride (30 mM in methanol), and solutions of chlorobenzene and 2-chloro-2-methylpropane (100 mM in methanol) were prepared in a nitrogen glovebox for each experiment. Solutions of Eu^{II}**1** were prepared by combining equal volumes of solutions of **1** (60 mM) and $EuCl_2$ (60 mM) and stirring the resulting solution for one hour. Benzyl chloride, allyl chloride, chlorobenzene, and 2-chloro-2-methylpropane were weighed by dropping neat substrate from a syringe into a vial tared with 2 mL of methanol, and diluting with methanol to a final concentration of either 30 or 100 mM.

Cuvettes were prepared by mixing solutions in accordance with the corresponding table for each substrate (Tables S3–S6). The caps of the cuvettes were sealed with electrical tape before being transported from the glovebox in a beaker covered in foil. Stern–Volmer analysis of benzyl chloride was performed on three independent sets of cuvettes. Plotted points and error bars represent the average of those analyses and standard deviation, respectively.

Steady-state emission spectra were collected with a Jobin Yvon Fluoromax-4P spectrophotometer with λ_{ex} = 460 nm with 1 nm slit width and λ_{em} = 470–800 nm with 1 nm slit width and 1 nm resolution for all samples. Integration was performed over the entire range in all cases.

Table S3 Benzyl chloride cuvette preparation.

Sample	Eu**1**^{II}Cl$_2$, 30 mM (mL)	Benzyl chloride, 30 mM (mL)	Methanol (mL)	Total volume (mL)
0	0.2	0	2.8	3
1	0.2	0.5	2.3	3
2	0.2	1.0	1.8	3
3	0.2	1.5	1.3	3

Table S4 Allyl chloride cuvette preparation.

Sample	Eu**1**^{II}Cl$_2$, 30 mM (mL)	Allyl chloride, 30 mM (mL)	Methanol (mL)	Total volume (mL)
0	0.2	0	2.8	3
1	0.2	0.5	2.3	3
2	0.2	1.0	1.8	3
3	0.2	1.5	1.3	3

Table S5 Chlorobenzene cuvette preparation.

Sample	Eu**1**^{II}Cl$_2$, 30 mM (mL)	Chlorobenzene, 100 mM (mL)	Methanol (mL)	Total volume (mL)
0	0.2	0	2.8	3
1	0.2	0.4	2.4	3
2	0.2	0.9	1.9	3

Table S6 2-chloro-2-methylpropane cuvette preparation.

Sample	Eu**1**^{II}Cl$_2$, 30 mM (mL)	2-chloro-2-methylpropane, 100 mM (mL)	Methanol (mL)	Total volume (mL)
0	0.2	0	2.8	3
1	0.2	0.4	2.4	3
2	0.2	0.9	1.9	3

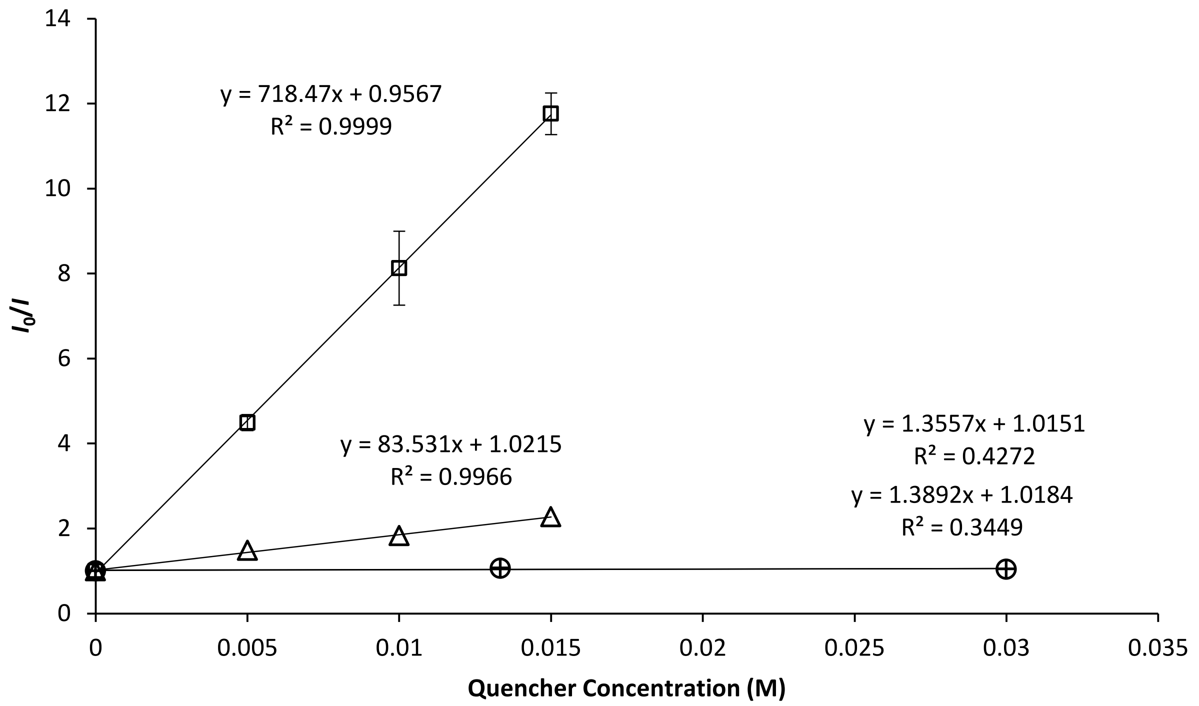

Figure S15 Ambient temperature Stern–Volmer analyses with benzyl chloride (squares), allyl chloride (triangles), chlorobenzene (circles), and 2-chloro-2-methylpropane (plus signs). Error bars represent the standard deviation of the mean of the measurements of independently prepared samples.

Variable-temperature Stern–Volmer

Cuvettes were prepared in accordance with Table S3 in a dry glovebox independently of the ambient temperature studies. These cuvettes were sealed with paraffin wax. Emission data was collected in the same way as for ambient temperature Stern–Volmer analyses. After the initial ambient temperature data was collected, each sample was placed in a water bath (50 ^{o}C) covered in aluminum foil for 30 min and then measured again. The cuvettes were then placed in a foil-covered beaker at ambient temperature for 15 min, transferred to an ice water bath in the dark for 30 min, and then measured again. Each sample was then placed in a foil-covered beaker at ambient temperature for 30 min and measured again. Data from these measurements are shown in Figure S16.

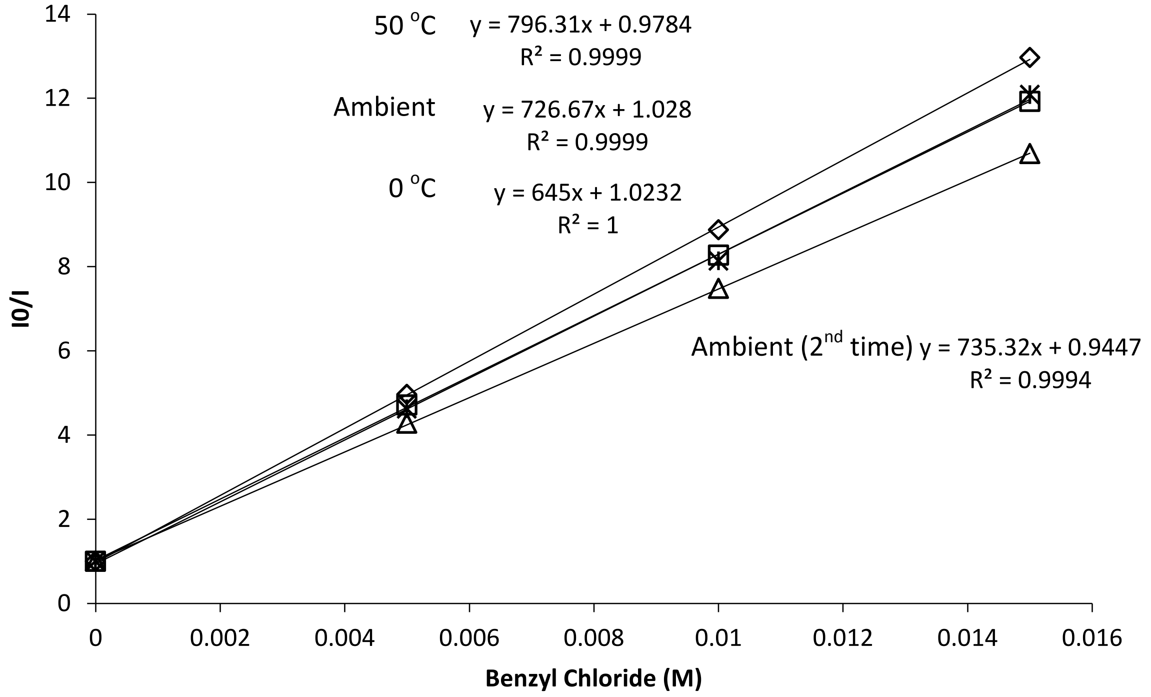

Figure S16 Variable temperature Stern–Volmer analysis: initial ambient-temperature measurements (squares), 50 °C measurements (diamonds), 0 °C measurements (triangles), and final ambient-temperature measurements (asterisks).

Eu^{III} Binding Study

Solid **1** (32.8 mg, 88.5 mmol) and $EuCl_3{\cdot}6H_2O$ (62.0 mg, 169.2 mmol) were weighed into separate 10 mL vials. To the vial of **1** was added methanol (6.0 mL) for a final concentration of **1** of 15 mM. To the vial of $EuCl_3{\cdot}6H_2O$ was added methanol (5.5 mL) for a final concentration of $EuCl_3{\cdot}6H_2O$ of 30 mM. To create a solution of $EuCl_3{\cdot}6H_2O$ (10 mM in methanol), the aforementioned $EuCl_3{\cdot}6H_2O$ (1.0 mL) was added to a quartz cuvette followed by methanol (2.0 mL). This solution was agitated by hand for 30 s. An emission spectrum was acquired of the solution using a Jobin Yvon Fluoromax-4P spectrophotometer (λ_{ex} = 395 nm with 3 nm slit width, λ_{em} = 550 to 680 nm with 1 nm slit width and 0.1 nm resolution, ··· in Figure S17).

To create a solution of both **1** (10 mM) and $EuCl_3{\cdot}6H_2O$ (10 mM), an aliquot of $EuCl_3{\cdot}6H_2O$ (1.0 mL, 30 mM) was added to a cuvette followed by an aliquot of **1** (2.0 mL, 15 mM). A Teflon stir flea was added to the cuvette. The solution was stirred for 30 min and an emission spectrum was acquired with the same parameters as the $EuCl_3{\cdot}6H_2O$ methanol solution (–·– in Figure S17).

A blank sample from a Stern–Volmer experiment (2 mM of Eu^{II}**1**, no quencher) was opened to air for 30 min, capped, and agitated by hand. An emission spectrum was acquired with the same parameters as the $EuCl_3{\cdot}6H_2O$. This spectrum shows the same profile as that of $EuCl_3{\cdot}6H_2O$ mixed with **1** (— in Figure S17).

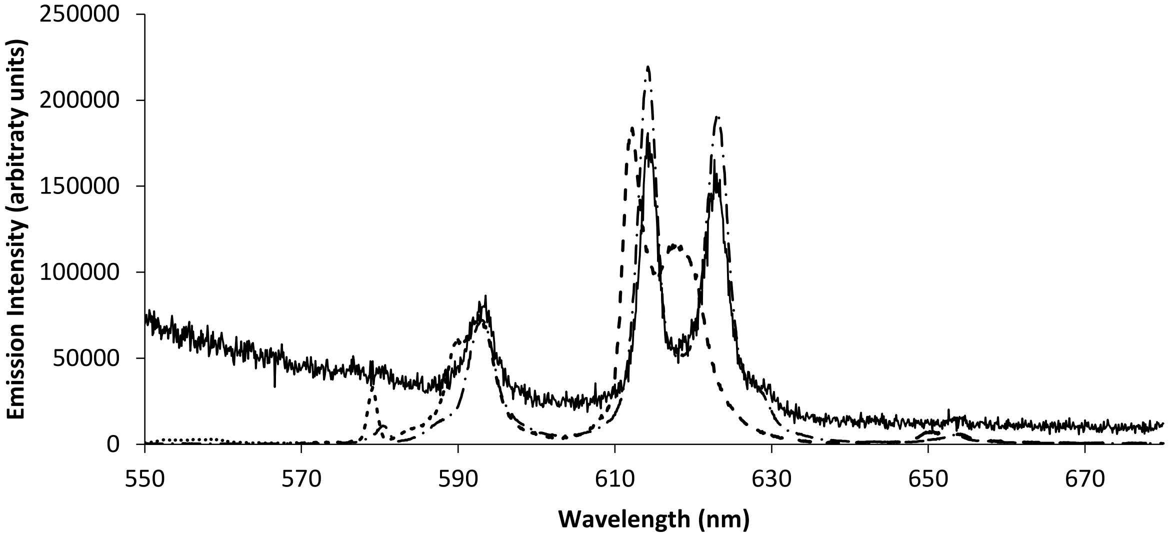

Figure S17 Eu^{III} binding study. $EuCl_3$ in methanol (10 mM, ···), $EuCl_3$ and **1** (10 mM each, –·–), and oxidized Eu^{II}**1** (2 mM, —).

UV–visible spectrum of zinc-reduced Eu^{II}**1**

To a 20 mL vial in a dry glovebox containing **1** (10.1 mg, 0.0272 mmol), $EuCl_3$ (8.7 mg, 0.039 mmol), and zinc dust (187.0 mg, 28.60 mmol) was added methanol (3 mL), and the resulting mixture was stirred for 2 h. The solution was filtered into a new 20 mL vial through a 0.22 µm hydrophilic filter. A cuvette was prepared with the aforementioned filtered solution (0.2 mL) and fresh methanol (2.8 mL). The cuvette was sealed with electrical tape and removed from the glovebox. A Shimadzu UVmini-1240 spectrophotometer was baseline corrected with a cuvette of methanol (3 mL) and a spectrum of the aforementioned filtered solution was collected from 190 to 1100 nm with a 1 nm resolution (Figure S18).

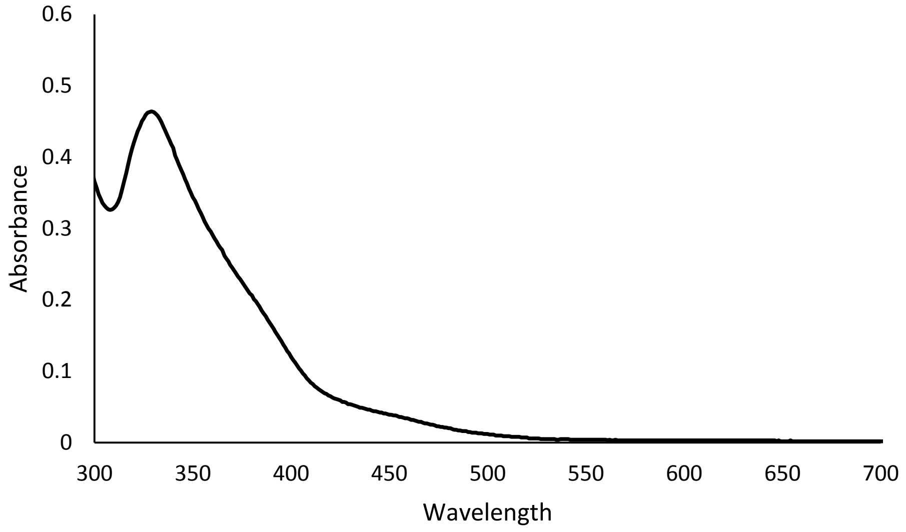

Figure S18 UV–visible spectrum of *in situ* generation of Eu^{II}**1**.

Luminescence spectrum of zinc-reduced Eu^{II}**1**

To obtain an emission spectrum of zinc-reduced Eu^{II}**1**, the same sample was used from the UV–visible spectrum of zinc-reduced Eu^{II}**1** experiment. Emission was collected on a Jobin Yvon Fluoromax-4P spectrophotometer (λ_{ex} = 460 nm with 1 nm slit width, λ_{em} = 465 to 900 nm with a 1 nm slit width at 1 nm resolution Figure S19).

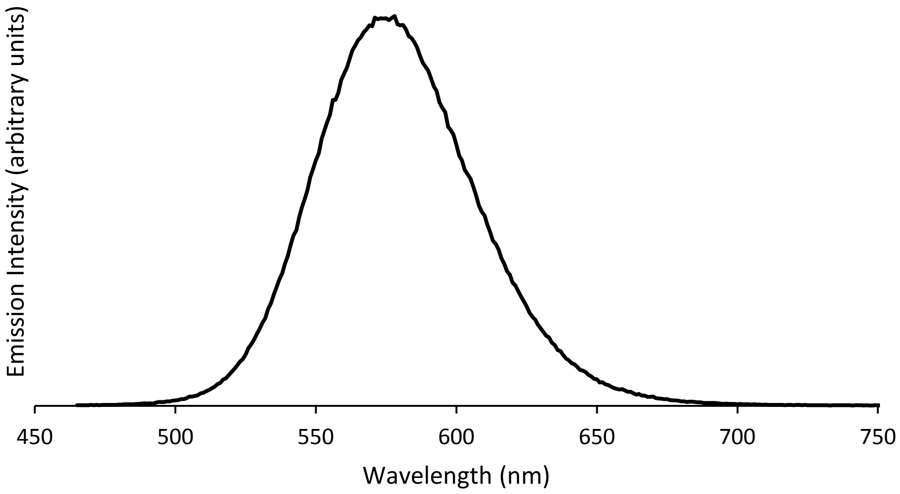

Figure S19 Fluorescence spectrum of *in situ* generation of Eu^{II}**1**, λ_{ex} = 460 nm.

Emission spectrum of methanolic Eu^{II}1 after 12 h in either darkness or a photoreactor.

Immediately after quantum yield data were acquired, sample 3 from the quantum yield experiment was placed in a photoreactor for 12 h, and sample 4 from the quantum yield experiment was placed in a foil covered beaker for 12 h. Emission spectra were collected using a Jobin Yvon Fluoromax-4P spectrophotometer (λ_{ex} = 460 with a 1 nm slit width, and λ_{em} = 470 to 800 nm with a 1 nm slit width at 1 nm resolution for each solution Figure S20).

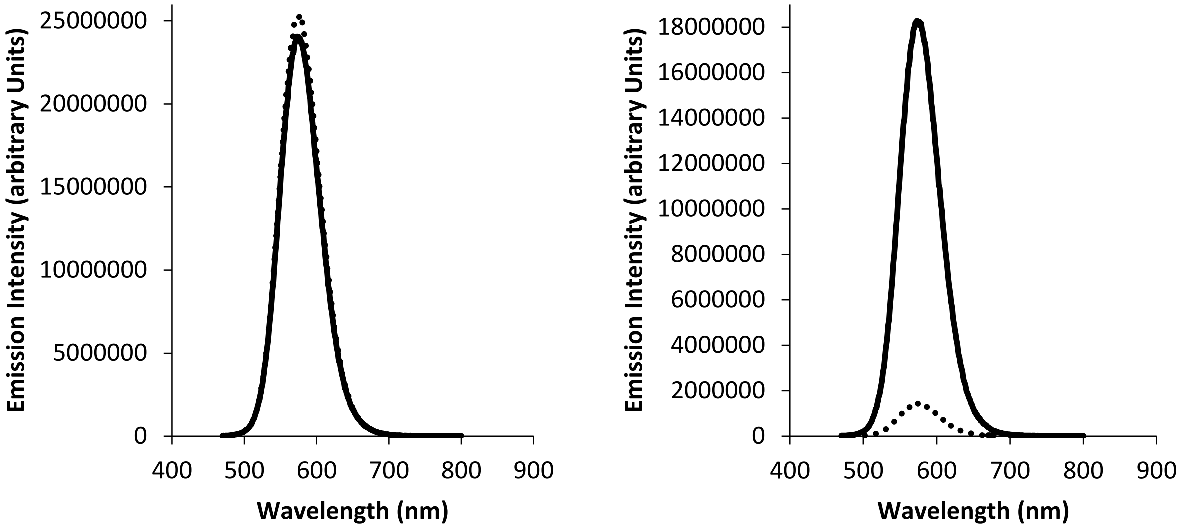

Figure S20 Emission before (—) and after (···) 12 h of either darkness (left) or exposure to blue LED light in a photoreactor (right).

Lifetime Study of methanolic Eu^{II}1

Lifetime measurements were obtained by use of a Photonics DM-20 pump laser in conjunction with a Red Dragon™ ultrafast 800 nm Ti-Sapphire laser amplifier. The 800 nm fundamental laser beam was upconverted using an SHG crystal (BBO) and passed through a 10 mm^2 cuvette containing a solution of Eu^{II}**1** (2 mM). Luminescence signal was detected with a photodiode with attached wavelength filter placed at 90° from the incident beam and obtained using a RIGOL-DS1302CA digital oscilloscope running at average mode. Fitting of the experimental data was performed using a double exponential decay fitting using MATLAB. Three measurements were acquired, at three different intensities of the incident beam.

Two lifetime values were obtained from this experiment, 0.98 ± 0.03 and 23 ± 6 μs. The former, shorter lifetime does not vary with incident beam intensity and is the predominant contribution to the decay profile, which we assigned to be the lifetime of Eu^{II}**1**. The latter decay lifetime varies with incident beam intensity and is possibly due to one or more secondary effects such as multi-photon excitation, intersystem crossing producing different excited states, or instrument response of the experimental setup. An example fitting is shown in Figure S21.

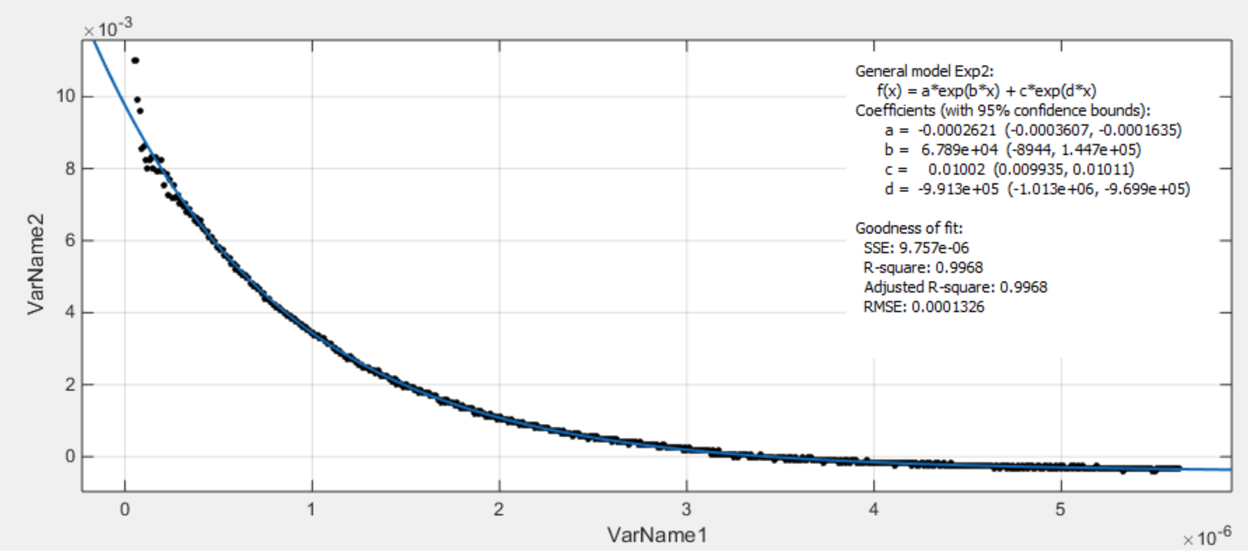

Figure S21 Representative spectrum of lifetime fitting for Eu^{II}**1**. The x-axis is time in units of seconds (note the factor of 10^{-6}), and the y-axis is emission intensity (arbitrary units).

References

1 (*a*) M. Y. Redko, R. Huang, J. L. Dye and J. E. Jackson, *Synthesis*, 2006, **5**, 759; (*b*) P. H. Smith, M. E. Barr, J. R. Brainard, D. K. Ford, H. Freiser, S. Muralidharan, S. D. Reilly, R. R. Ryan, L. A. Silks, III and W.-H. Yu, *J. Org. Chem.*, 1993, **58**, 7939.

2 G. M. Sheldrick, *Acta Cryst.*, 2015, **A71**, 3.

3 G. M. Sheldrick, *Acta Cryst.*, 2008, **A64**, 112.

4 C. B. Hübschle, G. M. Sheldrick and B. Dittrich, *J. Appl. Cryst.*, 2011, **44**, 1281.

5 (*a*) A. M. Brouwer, *Pure Appl. Chem.*, 2011, **83**, 2213; (*b*) C. Würth, M. Grabolle, J. Pauli, M. Spieles and U. Resch-Genger, *Anal. Chem.*, 2011, **83**, 3431.

6 (*a*) J. W. Tucker and C. R. J. Stephenson, *J. Org. Chem.*, 2012, **77**, 1617; (*b*) D. Rehm and A. Weller, *Isr. J. Chem.*, 1970, **8**, 259.

7 *CRC Handbook of Chemistry and Physics*, 1st Student Edition (Ed.: R. C. Weast), CRC PRESS, Inc., Boca Raton, 1988, p. E-316.
